# The Level of Ets-1 Protein Is Regulated by Poly(ADP-Ribose) Polymerase-1 (PARP-1) in Cancer Cells to Prevent DNA Damage

**DOI:** 10.1371/journal.pone.0055883

**Published:** 2013-02-06

**Authors:** Arnaud J. Legrand, Souhaila Choul-Li, Corentin Spriet, Thierry Idziorek, Dorothée Vicogne, Hervé Drobecq, Françoise Dantzer, Vincent Villeret, Marc Aumercier

**Affiliations:** 1 CNRS USR 3078, Institut de Recherche Interdisciplinaire, Campus CNRS de la Haute Borne, Université Lille Nord de France, IFR 147, BP 70478, Villeneuve d'Ascq, France; 2 CNRS UMR 8161, Institut de Biologie de Lille, Institut Pasteur de Lille, Universités Lille Nord de France, IFR 142, BP 447, Lille, France; 3 Inserm U837, Institut de Recherche sur le Cancer de Lille, Université Lille-Nord de France, IFR 114, Lille, France; 4 CNRS UMR 7242, Institut de Recherche de l'Ecole de Biotechnologie de Strasbourg, Ecole Supérieure de Biotechnologie de Strasbourg, Université de Strasbourg, BP 10413, Illkirch, France; UAE University, United Arab Emirates

## Abstract

Ets-1 is a transcription factor that regulates many genes involved in cancer progression and in tumour invasion. It is a poor prognostic marker for breast, lung, colorectal and ovary carcinomas. Here, we identified poly(ADP-ribose) polymerase-1 (PARP-1) as a novel interaction partner of Ets-1. We show that Ets-1 activates, by direct interaction, the catalytic activity of PARP-1 and is then poly(ADP-ribosyl)ated in a DNA-independent manner. The catalytic inhibition of PARP-1 enhanced Ets-1 transcriptional activity and caused its massive accumulation in cell nuclei. Ets-1 expression was correlated with an increase in DNA damage when PARP-1 was inhibited, leading to cancer cell death. Moreover, PARP-1 inhibitors caused only Ets-1-expressing cells to accumulate DNA damage. These results provide new insight into Ets-1 regulation in cancer cells and its link with DNA repair proteins. Furthermore, our findings suggest that PARP-1 inhibitors would be useful in a new therapeutic strategy that specifically targets Ets-1-expressing tumours.

## Introduction

Ets-1 is the founding member of the family of transcription factors called ETS. This family is characterised by a well-conserved DNA-binding domain (DBD)^5^ that recognises specific DNA elements, called ETS-binding sites (EBS), found in the promoters of target genes. Ets-1 is mainly expressed in embryonic tissues. It is involved in physiological processes such as proliferation, differentiation, migration, invasion and apoptosis [1]–[6]. Ets-1 expression is tightly regulated in adult tissues and its overexpression is often related to invasive diseases, such as rheumatoid arthritis, glomerulonephritis and many cancers [7]–[9]. The pathological expression of Ets-1 is partly responsible for the proliferation and invasion abilities of tumour cells. This invasiveness is due to genes that are controlled by Ets-1 and that encode proteases, including the matrix metalloproteases collagenase-1 and stromelysin-1, or the urokinase-type plasminogen activator (uPA). Therefore, Ets-1 is currently considered as a marker of poor prognosis in several cancers [10]–[13].

Moreover, despite the fact that all ETS family members share the same DBD, Ets-1 has its own DNA-binding properties that are tightly controlled to ensure a specific biological action. Ets-1 inhibits its own DNA binding due to the presence of two inhibitory domains that flank its DBD [14]. Ets-1 needs to interact with partners to counteract this auto-inhibition and bind to the promoters of its target genes [15]. In some promoters, such as those found in the *MMP3* (stromelysin-1) and *TP53* genes, two Ets-1 molecules bind to two palindromic EBS separated by 4 bp [16]–[18]. In all cases, protein-protein interactions appear to be the keystone of Ets-1 regulation.

Furthermore, Ets-1 is also tightly controlled by post-translational modifications, including phosphorylation, SUMOylation and ubiquitination [7], [19]. However, Ets-1 shares the same signalling pathways with many transcription factors. Thus, the challenge is to identify the specific features of the pathways that control the activity of Ets-1 to use them for therapeutic targeting.

To identify novel pathways, we previously purified interaction partners of Ets-1 using the streptavidin pull-down assay. With this strategy, we have demonstrated the functional interaction between Ets-1 and the DNA repair complex DNA-PK [20].

Here, we identified poly(ADP-ribose) polymerase-1 (PARP-1) as a novel interaction partner of Ets-1. PARP-1 is an abundant and ubiquitous nuclear protein that catalyses poly(ADP-ribosyl)ation (PARylation) by using NAD+ as substrate to synthesise branched poly(ADP-ribose) polymers on target proteins. PARP-1 plays diverse roles in many molecular and cellular processes, including DNA damage detection and repair and chromatin modification [21]. Although PARP-1 was originally characterised as a DNA repair protein, many recent studies have highlighted its role in transcriptional regulation. PARP-1 is involved in the regulation of transcription factors such as NF-κB, AP-2, p53 and many others [22]–[24]. Furthermore, PARP-1 inhibition has emerged as a new therapeutic strategy for cancer [25]. Interestingly, PARP-1 inhibition seems to be effective in cancers that often show overexpressed ETS proteins, including ovarian, prostate and breast cancers [25]. Previous studies have shown a functional link between PARP-1 and ETS family members, such as Ese-1, Fli1, Erg and Elk-1 [26]–[28]. Likewise, Ets-1 may be a key regulator of the *parp1* gene by controlling its promoter [29]. Very recently, interest in this functional link has heightened to the extent that it is now considered as a brand-new therapeutic approach in cancer. Recent reports have demonstrated that ETS fusion proteins, which are involved in many cancers, are drug-sensitivity biomarkers of PARP-1 inhibition [26], [30], [31]. High expression of TMPRSS2:Erg in prostate cancers or EWS-Fli1 in Ewing sarcoma causes DNA damages that are potentiated by PARP-1 inhibition and are followed by strong inhibition of cancer progression. Nevertheless, as far as we know, there have been no reports of any functional links between Ets-1 expression and its sensitivity to PARP-1 inhibitors.

In this study, we demonstrate that PARP-1 negatively controls the level of Ets-1 proteins in cancer cells via PARylation. Under PARylation inhibition, Ets-1 transcriptional activity is enhanced which correlates with Ets-1 proteins accumulation in cell nuclei and an increase in DNA damage that leads to cancer cell death. Therefore, our results suggest that the inhibition of PARylation could be a novel therapeutic strategy for limiting cancer progression in Ets-1-expressing tumours.

## Results

### PARP-1: a novel Ets-1 interaction partner in cancer cells

**Figure 1:**
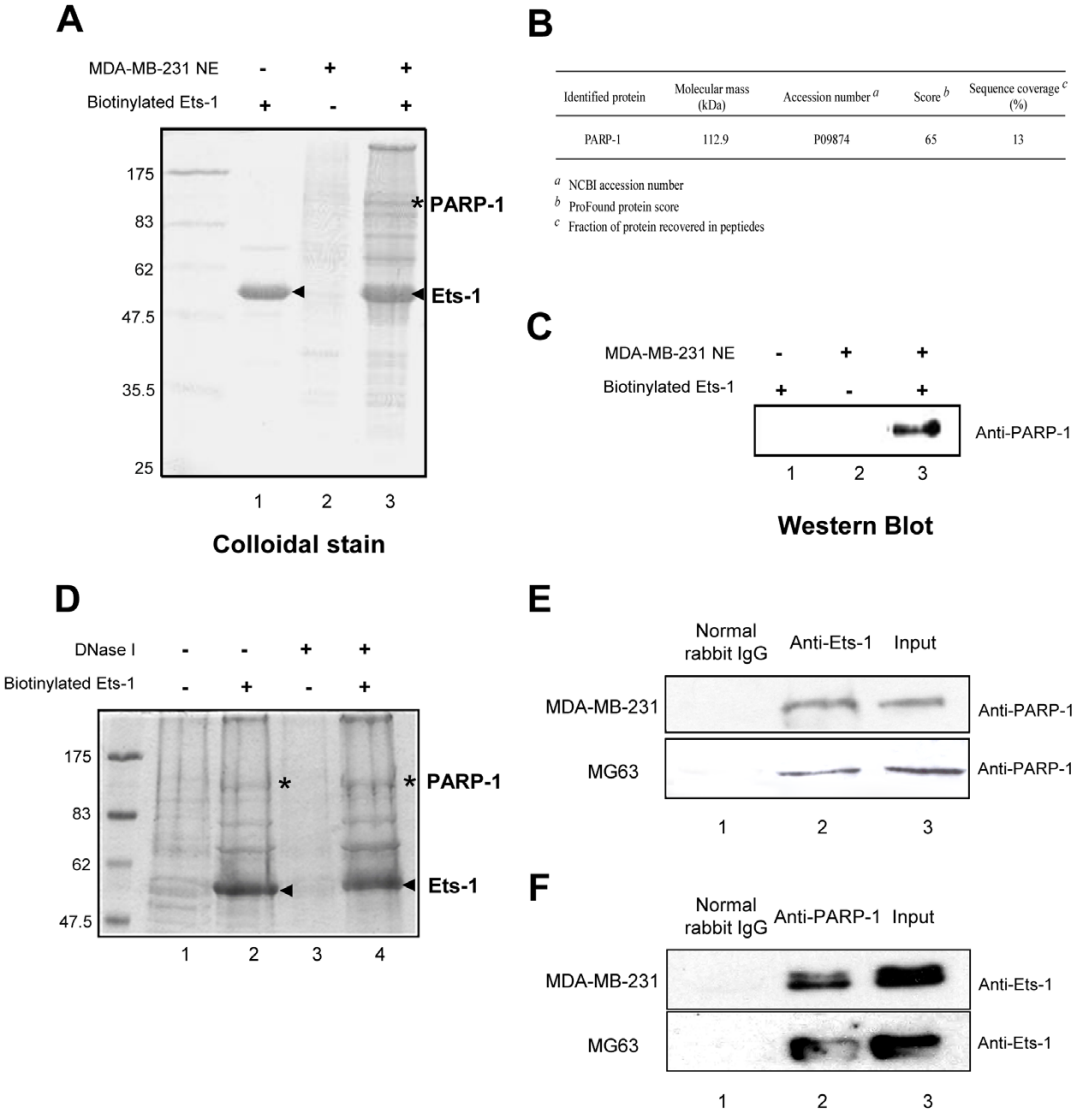
PARP-1 is a novel interaction partner of Ets-1. (**A**) Colloidal blue-stained gel for Ets-1-associated proteins from MDA-MB-231 nuclear extracts. Biotinylated Ets-1 proteins used for the pull-down assay were loaded alone as a control to identify the biotinylated proteins (arrowheads) and their potential breakdown products (lane 1). Pull-down proteins detected from nuclear extracts (NE) incubated with unloaded beads were considered to be non-specific products (lane 2). The colloidal blue-stained specific PARP-1 protein pulled-down by biotinylated Ets-1 is indicated by an asterisk (lane 3). (**B**) Identification of PARP-1 by MALDI-TOF mass spectrometry. (**C**) Western blot analysis confirming the Ets-1-associated protein as PARP-1. Proteins pulled-down from MDA-MB-231 nuclear extracts were analysed by Western blot with rabbit antibodies directed against PARP-1 (H-250). (**D**) Colloidal blue-stained gel for Ets-1-associated proteins from MDA-MB-231 nuclear extracts either untreated (lanes 1 and 2) or treated with DNase I for 40 min (lanes 3 and 4) and then either incubated with streptavidin beads loaded with biotinylated Ets-1 protein (arrowheads) (lanes 2 and 4) or with unloaded beads (lanes 1 and 3). Asterisks indicate the PARP-1 protein detected in (A). (**E**) and (**F**) Co-immunoprecipitation performed using a rabbit anti-Ets-1 (C-20) or a rabbit anti-PARP-1 (H-250) antibody-agarose conjugate (lane 2) or normal rabbit IgG as a control (lane 1) and nuclear extracts from MDA-MB-231 cells or MG-63 cells. Input (lane 3) contains 3% of nuclear extracts that underwent co-immunoprecipitation. PARP-1 and Ets-1 were analysed by Western blot using respectively H-250 and C-4 antibodies.

To find novel Ets-1 partners, we carried out an *in vitro* affinity purification strategy using a streptavidin pull-down assay. This approach ensures large-scale identification of partners by using biotinylated recombinant Ets-1 immobilised on streptavidin beads to retain its binding proteins found in nuclear extracts [20], [32]. Here, we used MDA-MB-231 cells, an invasive breast cancer cell line, to produce nuclear extracts; these cells endogenously express the Ets-1 oncoprotein, thereby offering an appropriate cellular context. After separation of the bound proteins by SDS-PAGE, a highly visible protein band with a molecular weight of 113 kDa was observed after colloidal staining (Fig. 1A, lane 3, indicated by an asterisk (*)), but absent in control conditions (Fig. 1A, lanes 1 and 2). This protein was identified by mass spectrometry as the PARP-1 enzyme (Fig. 1B and S1) and its exclusive presence in Ets-1-bound proteins was confirmed by Western blot (Fig. 1C, lane 3). Owing to the strong affinity of PARP-1 for nicked DNA, nuclear extracts were treated with DNase I before incubation to remove all traces of nucleic acids. This treatment did not disrupt the interaction between Ets-1 and PARP-1 (Fig. 1D, lane 4). This was confirmed by mass spectrometry (data not shown). We then performed co-immunoprecipitation experiments using an anti-Ets-1 antibody-agarose conjugate in MDA-MD-231 cells and in an osteosarcoma cell line, MG63, which also expresses Ets-1. After separation by SDS-PAGE and immunoblotting with an anti-PARP-1 antibody, PARP-1 co-precipitated with Ets-1 from both MDA-MB-231 and MG63 cells (Fig. 1E, lane 2). Control experiments with IgG from normal rabbit serum failed to recruit PARP-1 (Fig. 1E, lane 1). Reciprocal co-immunoprecipitation experiments were performed using an anti-PARP-1 antibody-agarose conjugate in MDA-MD-231 and MG63 cells. Ets-1 co-precipitated from both MDA-MB-231 and MG63 cells and was absent from control experiments (Fig. 1F). Furthermore, immunofluorescence experiments using anti-Ets-1 and anti-PARP-1 antibodies showed that both proteins co-localise in MDA-MB-231 cells nuclei (Fig. S2, lane 4).

### Ets-1 directly interacts with PARP-1 and is then PARylated in a DNA-independent manner

**Figure 2:**
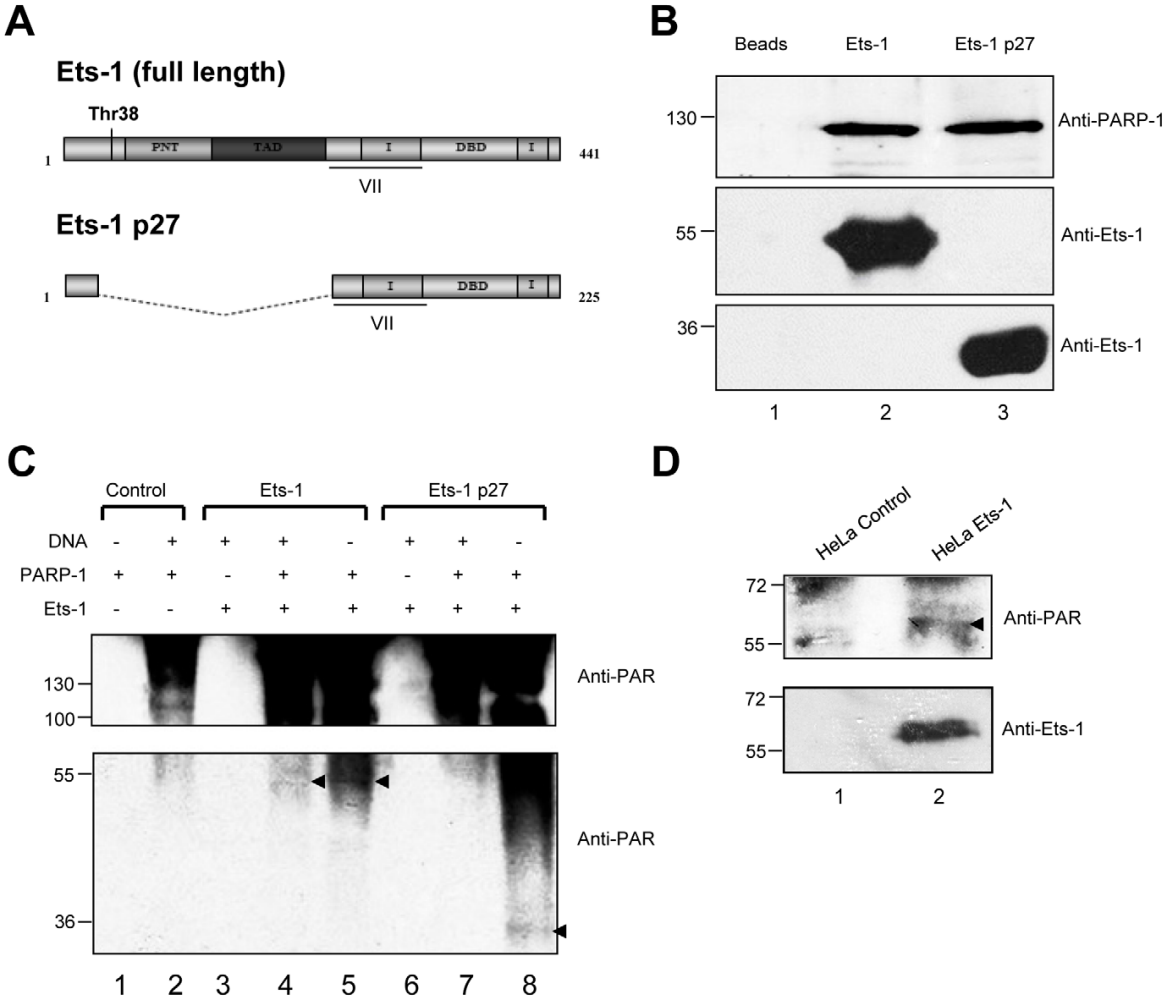
Ets-1 interacts directly with PARP-1 and is then poly(ADP-ribosyl)ated. (**A**) Schematic representation of the full-length Ets-1 protein and its dominant-negative isoform Ets-1 p27. Boxes correspond to translated regions and dotted lines to untranslated regions. Threonine-38 (Thr38), Pointed domain (PNT), transactivation domain (TAD), inhibitory domain (I), translated region corresponding to the exon VII and DNA-binding domain (DBD) are indicated. (**B**) Streptavidin pull-down assay with recombinant Ets-1 and PARP-1 proteins. Biotinylated Ets-1 (5 µg) or Ets-1 p27 (2.5 µg) proteins were loaded on streptavidin beads and then incubated with pure recombinant PARP-1 protein (1 µg) for 1 h (lanes 2 and 3). Streptavidin beads alone incubated with PARP-1 were used as control (lane 1). PARP-1 was analysed by Western blot. (**C**) PARylation assay with Ets-1 and Ets-1 p27. Ets-1 (1 µg) or Ets-1 p27 (500 ng) proteins were incubated with recombinant PARP-1 protein (500 ng) in PARylation reaction buffer (see Materials and Methods) in presence (lanes 4 and 7) or in absence (lanes 5 and 8) of nicked DNA for 20 min. As a control, Ets-1 isoforms were incubated alone in reaction buffer and nicked DNA (lanes 3 and 6) and auto-activation of PARP-1 alone was checked with or without DNA (control, lanes 1 and 2) (**D**) PARylation of Ets-1 in stably transfected HeLa cells, obtained by retroviral infection. Ets-1 tagged with streptavidin binding peptide (SBP) was purified on stretavidin beads from HeLa cell nuclear extracts (lane 2). HeLa cells stably expressing only the SBP were used as control (lane 1). In (C) and (D), PARylation status was determined using an anti-PAR antibody.

A previous study demonstrated that the ETS DBD interacts with PARP-1 [30]; nevertheless, interactions with the Ets-1 DBD are restricted by Ets-1 auto-inhibition. Therefore, to study the interaction between Ets-1 and PARP-1, we used two biotinylated and auto-inhibited isoforms of Ets-1: the full-length form, simply called Ets-1 and a dominant-negative isoform resulting from alternative splicing, Ets-1 p27. Ets-1 p27 was recently discovered by our group and lacks the threonine-38 (Thr38) residue as well as the Pointed (PNT) and transactivation (TAD) domains (Fig. 2A), all of which are required for the transactivation of Ets-1 target genes. However, Ets-1 p27 still has the same DNA-binding properties as Ets-1 [33]. After incubation of biotinylated Ets-1 isoforms immobilised on streptavidin beads with a recombinant PARP-1 enzyme, PARP-1 specifically and directly interacted with Ets-1 isoforms (Fig. 2B, lanes 2 and 3). Thus, the auto-inhibition of Ets-1 does not disrupt the direct interaction between the DBD and PARP-1. To investigate if Ets-1 is post-translationally modified by PARP-1-mediated PARylation, we carried-out an *in vitro* PARylation assay. To catalyse PARylation, PARP-1 generally requires the presence of nicked DNA, as shown in controls with the addition of sonicated double-stranded DNA (Fig. 2C lanes 1 and 2). Once activated, PARP-1 catalyses its auto-PARylation, which can be visualised using an anti-PAR antibody (Fig. 2C, lane 2). In the presence of Ets-1, auto-PARylation was stronger and Ets-1 was PARylated at a molecular weight of approximately 51 kDa corresponding to mono(ADP-ribosyl)ation and PARylation with different polymer sizes (Fig. 2C, lane 4, black arrow). Furthermore, in the absence of DNA, not only was PARP-1 still activated, but Ets-1 was even more strongly PARylated (Fig. 2C, lane 5, black arrow). With the Ets-1 p27 isoform, results showed that in presence of nicked DNA, Ets-1 p27 was not PARylated (Fig. 2C, lane 7). Nevertheless, with no DNA, Ets-1 p27 activates PARP-1 and is then PARylated, seen as a band at a molecular weight of approximately 27 kDa and with different sized polymers at molecular weights between 40 and 60 kDa (Fig. 2C, lane 8). Thus, the C-terminal extremity of Ets-1 can activate auto-PARylation of PARP-1 in a DNA-independent manner by direct protein-protein interaction and be PARylated in return. To demonstrate PARylation in human cancer cells, we generated a HeLa cell line, obtained by retroviral infection, that stably expresses Ets-1 tagged with a streptavidin-binding peptide (SBP). After purification of Ets-1 on streptavidin beads, we analysed PARylation by Western blot using an anti-PAR antibody. Results showed that a fraction of Ets-1 protein was slightly PARylated in cells (Fig. 2D, lane 2). PARylation is a very short-lived post-translational modification that is difficult to visualise in cells, particularly due to the action of the poly(ADP-ribose) glycohydrolase (PARG), which catalyses the degradation of PAR polymers [21].

### PARP-1 modulates Ets-1-mediated transcription of the stromelysin-1 promoter

**Figure 3:**
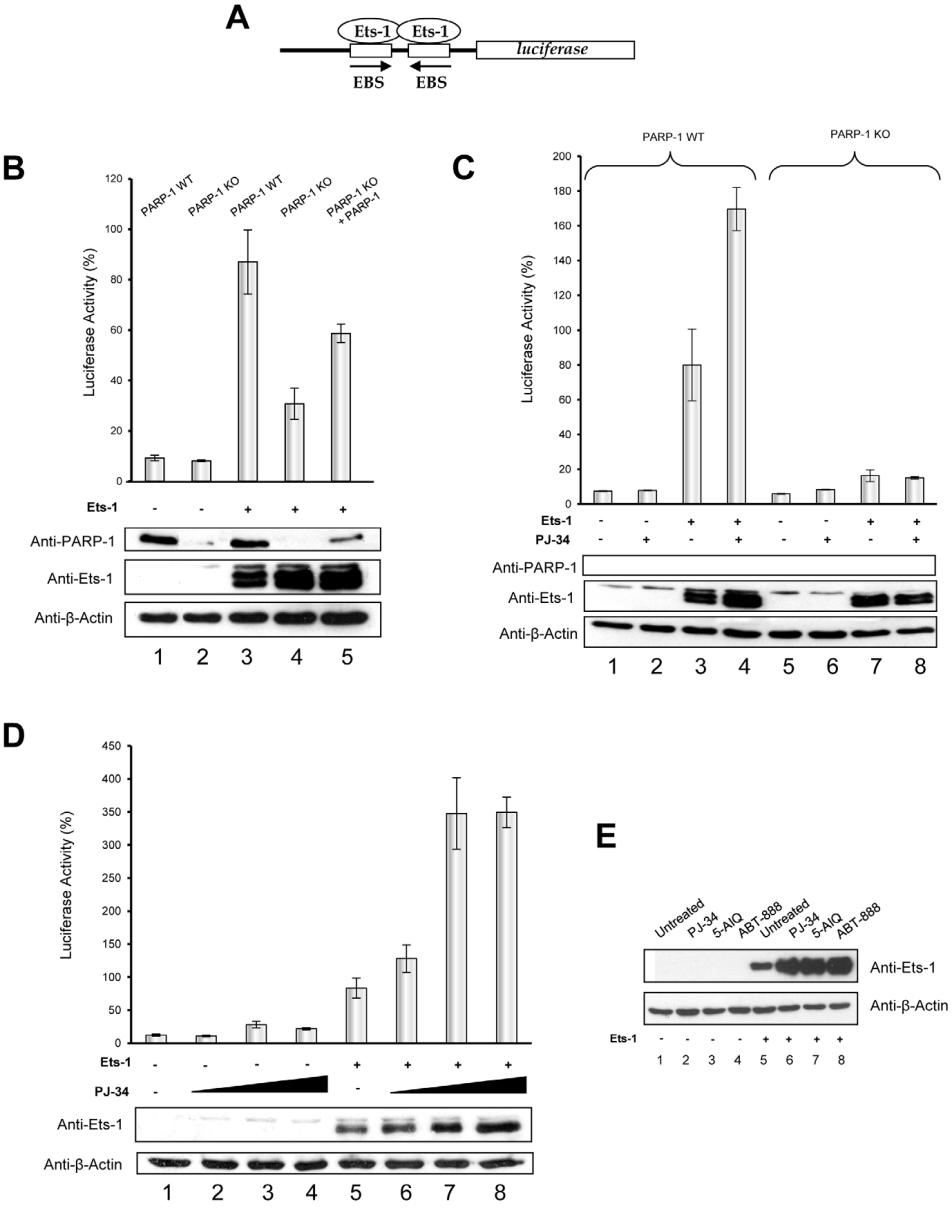
PARP-1 modulates Ets-1-mediated transactivation of the stromelysin-1 promoter by interaction and by PARylation. (**A**) Schematic representation of the luciferase gene under the control of the human stromelysin-1 promoter. Palindromic Ets-binding sites (EBS) are shown bound with two Ets-1 molecules. (**B**) Effect of PARP-1 knock out (KO) on Ets-1-mediated stromelysin-1 promoter activity. pGL3 luciferase reporter constructs (100 ng) were transfected into 3T3 mouse cells wild type (WT) (lanes 1 and 3) or KO for the PARP-1 gene (lanes 2, 4 and 5) at 50–80% confluence in the absence (lanes 1 and 2) or in the presence (lanes 3, 4 and 5) of the Ets-1 expression vector (25 ng) and in the rescue experiments with PARP-1 expression vector (100 ng; lane 5). (**C**) Effect of PARP-1 catalytic inhibition on the Ets-1-mediated stromelysin-1 promoter activity in the presence or absence of PARP-1. pGL3 luciferase reporter constructs (100 ng) were transfected into 3T3 mouse cells WT (lanes 1- 4) or KO for the PARP-1 gene (lane 5–8) at a 50–80% confluence in the absence (lanes 1, 2, 5 and 6) or in the presence (lanes 3, 4, 7 and 8) of Ets-1 expression vector (25 ng) and in the absence (lanes 1, 3, 5 and 7) or in the presence (lanes 2, 4, 6 and 8) of PJ-34, a catalytic inhibitor of PARP-1 (10 µM). (**D**) Effect of PARP-1 catalytic inhibition on Ets-1-mediated stromelysin-1 promoter activity in a cancer cell model. pGL3 luciferase reporter constructs (100 ng) were transfected into HeLa cells at a 50–80% confluence in the absence (lanes 1–4) or in the presence (lanes 5–8) of Ets-1 expression vector (100 ng) and with increasing doses of PJ-34 (0–20 µM; lanes 1–4 and 5–8). In (B), (C) and (D), luciferase activity, measured 48 h after transfection and 20 h after incubation with PJ-34, is expressed as a percentage with the activity induced by Ets-1 in a PARP-1 WT context indicated as 100%. Results are the average of three experiments performed in triplicate. (**E**) Effect of PARP-1 catalytic inhibition on the level of Ets-1 protein. HeLa cells were grown in 6-well plates until 70% confluence, transfected with pcDNA3 (1 µg; left panel) or pcDNA3-Ets1 (1 µg; right panel) vectors for 24 h and treated with PJ-34 (1 µM) (lanes 2 and 6), 5-AIQ (1 µM) (lanes 3 and 7) or ABT-888 (1 µM) (lanes 4 and 8) for 20 h. In all experiments, cell lysates (30 µg total proteins) were analysed by Western blot using different antibodies (see Materials and Methods).

To determine whether interaction with PARP-1 modulates the transcriptional activity of Ets-1, luciferase transactivation assays were performed. To do so, we used the promoter of the matrix metalloprotease stromelysin-1, which is a well-studied Ets-1 target gene involved in cancer cell migration and invasion [34]. This promoter, activated by cooperative binding of two Ets-1 molecules via a palindromic EBS, was fused with the luciferase gene (Fig. 3A). 3T3 mouse cells, with either the PARP-1 wild type (WT) or knock-out (KO), were used to evaluate Ets-1 transcriptional activity in total absence of the PARP-1 protein. In WT cells, the stromelysin-1 promoter was fully activated by Ets-1 transfection (Fig. 3B, lane 3). In contrast, PARP-1 KO reduced the ability of Ets-1 to transactivate the promoter (Fig. 3B, lane 4). Rescue experiments carried out using a human PARP-1 expression vector allowed Ets-1 to partly recover its transcriptional activity (Fig. 3B, lane 5). Thus, PARP-1 is necessary for full transactivation of the stromelysin-1 promoter by Ets-1. We then examined the consequences of PARP-1-mediated PARylation on Ets-1 transcriptional activity. To do so, we performed the same luciferase transactivation assays with 3T3 cells using PJ-34, a PARP-1 catalytic inhibitor. Surprisingly, PARylation inhibition, contrary to PARP-1 KO, increased Ets-1 transcriptional activity on the stromelysin-1 promoter as shown in transfected WT cells (Fig. 3C, lanes 3 and 4). In KO cells, PJ-34 had no effect and failed to increase transactivation of the promoter (Fig. 3C, lanes 7 and 8). Therefore, increases in Ets-1 transcriptional activity act through the specific inhibition of PARP-1. Analysis by Western blot revealed that inhibition of PARylation by PJ-34 caused an increase in Ets-1 protein level in WT cells (Fig. 3C, lanes 3 and 4). To confirm that PARylation inhibition also causes an increase in Ets-1 transcriptional activity in human cancer cells, we carried out luciferase transactivation assays in HeLa cells with increasing doses of PJ-34. Results showed that the PJ-34 dose-dependent increase in transactivation of the stromelysin-1 promoter was correlated with an increase in Ets-1 protein levels (Fig. 3D, lanes 5–8). This increase in Ets-1 protein levels was observed in HeLa cells not only with PJ-34 but also with two other well-studied PARP-1 inhibitors: 5-AIQ and ABT-888 (veliparib) (Fig. 3E, lanes 6–8). Thus, this increase is specifically due to PARP-1 inhibition. These results suggest that Ets-1 protein levels are negatively regulated by PARP-1-mediated PARylation.

### Inhibition of PARylation causes accumulation of Ets-1 in cancer cells

**Figure 4:**
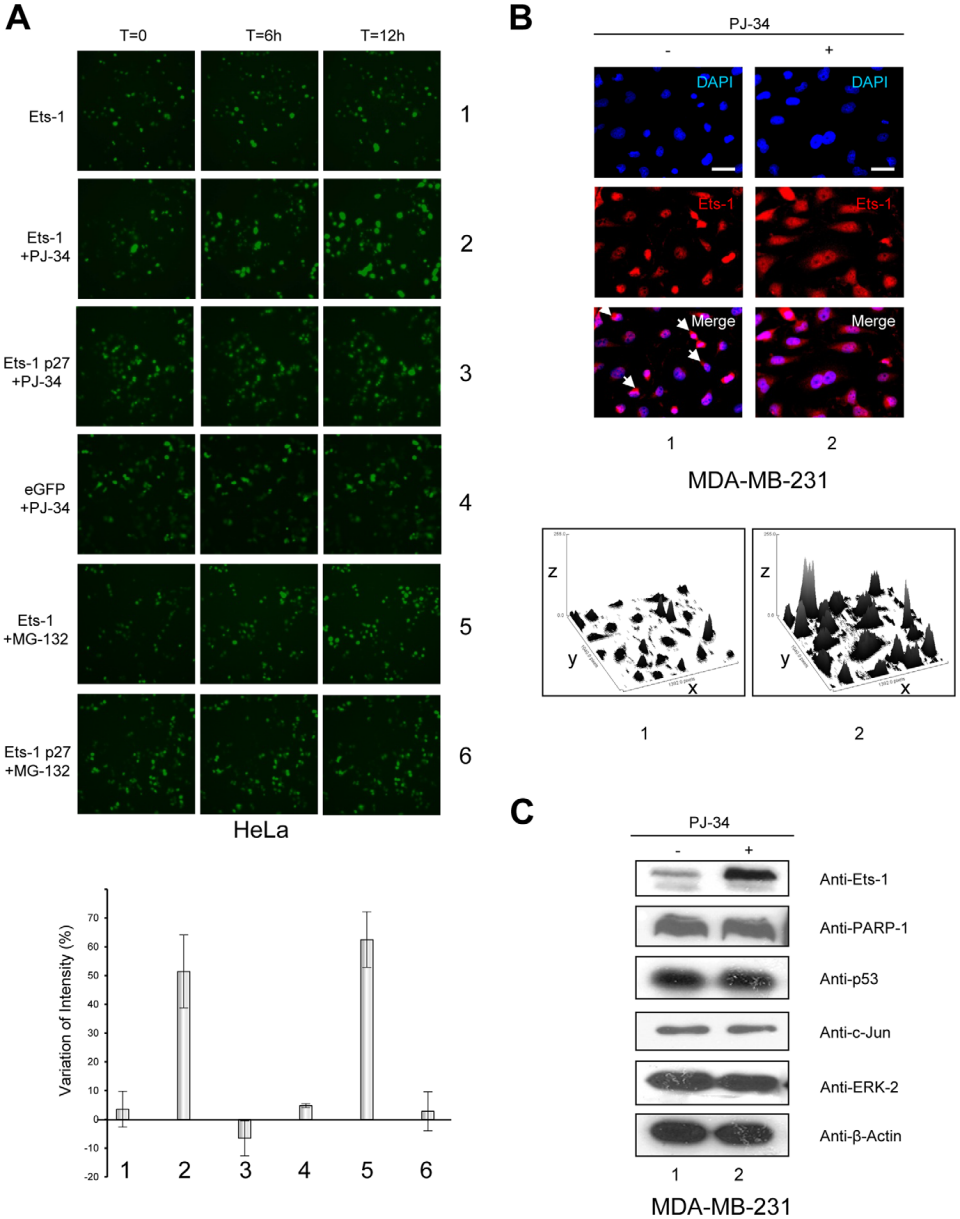
Inhibition of PARP-1-mediated PARylation induces the accumulation of Ets-1 protein. (**A**) Kinetics of Ets-1 accumulation observed by time-lapse imagery. HeLa cells were grown in MatTek dishes and were transfected with peGFP-C1-Ets-1 (500 ng; rows 1, 2 and 5) or peGFP-C-1-Ets-1p27 (500 ng; rows 3 and 6) or empty peGFP-C1 vectors (500 ng; row 4). 24 h after transfection, HeLa cells were treated with PJ-34 (10 µM; rows 2–4) or MG-132 (5 µM; rows 5 and 6) and observed for 12 h under a fluorescence microscope at ×20 magnification with one picture taken every hour. Bottom panel: Statistical analysis of the variation of fluorescence intensity. Conditions 1 to 6 indicated on the x-axis are the same as those described above. Mean fluorescence intensities from time-lapse experiment pictures were calculated using ImageJ software and the ratio of variation was established between the T = 12 h and T = 0 pictures. Results are the average of three experiments. (**B**) Visualisation of Ets-1 protein levels in MDA-MB-231 cells treated with PJ-34 by immunofluorescence. MDA-MB-231 cells were treated with PJ-34 (10 µM) for 20 h. Ets-1 is visualised in Red (Alexa Fluor® 594). Cells were examined under a fluorescence microscope at ×40 magnification. Scale bar = 20 µm. White arrows indicate cytoplasmic localisation of Ets-1 in untreated cells (lane 1). Bottom panel: Surface plot representations. Surface plots were obtained by analysing immunofluorescence pictures using ImageJ software; the x- and y-axes indicate the pixel positions and the z-axis, the intensity of fluorescence. (**C**) Effect of PARP-1 catalytic inhibition on the level of PARylated proteins. MDA-MB-231 cells were treated with PJ-34 (10 µM) for 20 h. Cell lysates (30 µg total proteins) were analysed by Western blot using different antibodies (see Materials and Methods) against PARP-1, p53, c-Jun and ERK-2 which are known to undergo PARylation.

To determine the kinetics of Ets-1 accumulation, we performed time-lapse imaging experiments. HeLa cells were transiently transfected by a vector expressing a fusion protein eGFP (enhanced green fluorescent protein)-Ets-1. Under control conditions, eGFP-Ets-1 protein levels were stable during a 12 h time-lapse (Fig. 4A, row 1). Upon addition of PJ-34, we observed a strong increase in the eGFP-Ets-1 signal corresponding to the nuclei of HeLa cells (Fig. 4A, row 2). Nevertheless, PJ-34 had no effect on eGFP alone (Fig. 4A, row 4) and, more interestingly, had no effect on eGFP-Ets-1 p27 (Fig. 4A, row 3). Given that full-length Ets-1 is ubiquitinated and then degraded by the proteasome, whereas Ets-1 p27 does not have any ubiquitination sites [19], [33], we next examined whether the kinetics of Ets-1 accumulation could be compared to those of proteasomal inhibition. Using MG-132, a well-known proteasome inhibitor, resulted in the same kinetics of Ets-1 accumulation as those observed during PARylation inhibition (Fig. 4A, row 5). In contrast, MG-132 had no effect on Ets-1 p27 protein levels, thereby strengthening the link between PARylation and proteasome degradation of Ets-1 (Fig. 4A, row 6). Statistical analysis of the variation in fluorescence intensity in these time-lapse experiments showed that the significant variation in Ets-1 protein levels (approximately 50% increase) observed upon addition of PJ-34 or MG-132 was comparable (Fig. 4A, bottom panel, lanes 2 and 5). To confirm that accumulation of Ets-1 mediated by PARP-1 inhibition could also be observed in an endogenous context, we treated MDA-MB-231 cells with PJ-34 and analysed Ets-1 protein levels by immunofluorescence. Ets-1 was mainly localised in the nucleus of MDA-MB-231 cells but its presence was also observed in the perinuclear cytoplasm (Fig. 4B, lane 1, white arrows) [33]. With an anti-Ets-1 antibody, we noticed a strong accumulation of endogenous Ets-1 in cells' nuclei and cytoplasm (Fig. 4B, lane 2). We then examined whether this mechanism was specific to Ets-1. To do so, we tested several proteins known to be PARylated such as p53, c-Jun, ERK-2 and PARP-1 [21], [23], [27], [35]. Results showed that, except for Ets-1, none of these proteins accumulated in cells under PARylation inhibition by PJ-34 (Fig. 4C). We also observed that Ets-1 effectively accumulated in MDA-MB-231 cells upon treatment with other PARP-1 inhibitors, 5-AIQ and ABT-888, whereas p53 protein levels remained unchanged (Fig. S3). Thus, PARP-1-mediated PARylation is involved in a specific regulation of Ets-1 protein levels in cancer cells and we assume that this mechanism is linked to its proteasomal degradation.

### Ets-1 expression leads to cancer cell death under PARylation inhibition

**Figure 5:**
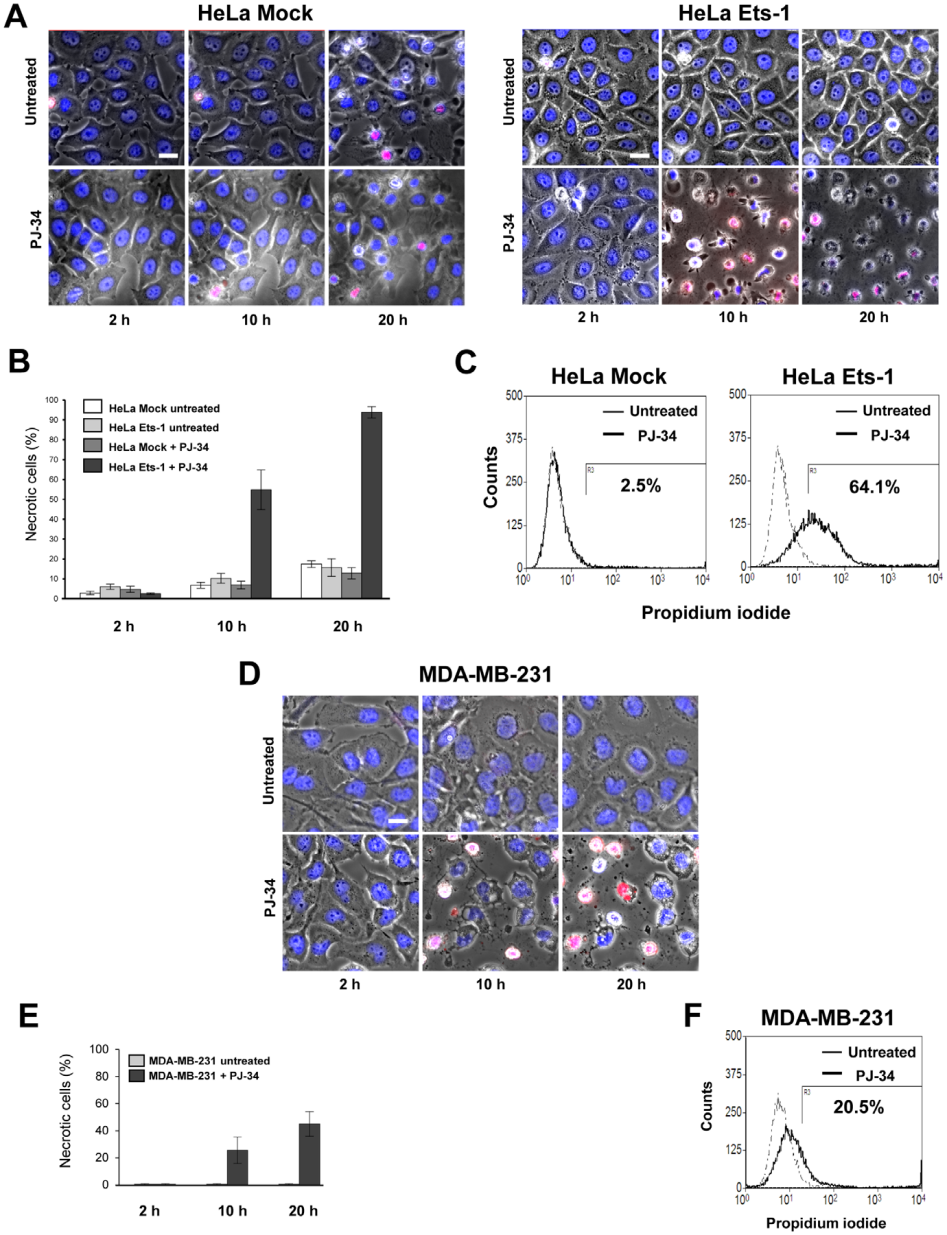
Ets-1 expression leads to cancer cell death under PARylation inhibition. (**A**) Time-lapse imaging experiments of HeLa cells treated with PJ-34. HeLa cells were grown in Hi-Q4 dishes until 70% confluence and transfected with empty pcDNA3 (250 µg; left panel) or pcDNA3-Ets1 (250 µg; right panel) vectors 24 h before being treated with PJ-34 (5 µM) or left untreated. Cells were stained with Hoechst 33242 (blue) and PI (red) for live-cell imaging and monitored for 20 h. Scale bar = 20 µM. (**B**) Graphical representation of the proportion of necrotic HeLa cells (%) at three time points (see Materials and Methods). (**C**) Flow cytometry cell-death detection of HeLa cells treated with PJ-34. HeLa cells were grown in 6-well plates until 70% confluence and transfected with pcDNA3 (1 µg; left panel) or pcDNA3-Ets1 (1 µg; right panel) vectors for 24 h and left untreated (dashed lines) or treated with PJ-34 (solid lines) for an additional 20 h incubation. Necrotic cell death was then determined by flow cytometry after PI staining. Numbers under the horizontal bar give the percentages of specific PJ-34-induced necrotic cell death in each condition. Flow cytometry profiles shown are representative of three replicate experiments. (**D**) Time-lapse imaging experiments of MDA-MB-231 cells treated with PJ-34. MDA-MB-231 cells were grown in Hi-Q4 dishes until 80% confluence and treated with PJ-34 (10 µM) or left untreated. Cells were stained with Hoechst 33242 (blue) and PI (red) for live-cell imaging and monitored for 20 h. Scale bar = 20 µM. (**E**) Graphical representation of the proportion of necrotic MDA-MB-231 cells (%) at three time points (see Materials and Methods). **F**) Flow cytometry cell-death detection of MDA-MB-231 cells treated with PJ-34. MDA-MB-231 cells were grown in 6-well plates until 80% confluence and left untreated (dashed lines) or treated with PJ-34 (solid lines) for a 20 h incubation. Necrotic cell death was then determined by flow cytometry after PI staining. Numbers under the horizontal bar represent the percentages of specific PJ-34-induced necrotic cell death in each condition. Flow cytometry profiles shown are representative of three replicate experiments.

To determine the consequences of Ets-1 accumulation in cancer cells, we examined whether exogenous Ets-1 expression affects the survival of HeLa cells treated with PJ-34. HeLa cells were transfected with a vector expressing Ets-1 or an empty vector and then incubated for 20 h with PJ-34. Time-lapse imaging experiments showed that PJ-34 did not have any drastic effect on the survival of HeLa cells transfected with the empty vector (‘Mock’) as demonstrated by the absence of necrotic morphology and propidium iodide (PI) incorporation (Fig. 5A, left panel and 5B). Nevertheless, Ets-1 expression sensitised cancer cells to PJ-34 toxicity to a great degree (Fig. 5A, right panel). Time-lapse imaging experiments and statistical analysis showed that almost all of the Ets-1-expressing HeLa cells incorporated PI and underwent necrosis (Fig. 5A, right panel and 5B). Similarly, a fluorescence-activated cell sorting (FACS) analysis demonstrated that Ets-1-expressing HeLa cells were more prone to necrosis after PJ-34 treatment (Fig. 5C, right panel). As PJ-34 is known to cause mitotic arrest in a PARP-1-independent manner [36], we also performed these experiments with ABT-888 (Fig. S4). The same results were obtained, demonstrating that the necrosis of Ets-1-expressing HeLa cells operates through specific inhibition of PARP-1 (Fig. S4). With MDA-MB-231 cells, we observed that 44% of the cells undergo to necrosis after PJ-34 treatment (Fig. 5D and 5E). FACS analysis confirmed this lower sensitivity to PJ-34 toxicity (Fig. 5F). Breast cancer cells are more resistant to anti-cancer drugs, such as doxorubicin or paclitaxel, due to high expression of MDR1 and Ets-1 [37]. Therefore, we tested whether PJ-34 could be used in complement to a doxorubicin treatment. These experiments also allowed us to investigate whether [or not] the Ets-1 accumulation mediated by PARP-1 inhibition causes a greater degree of resistance to doxorubicin in MDA-MB-231 cells. Results show that Ets-1 still accumulated under PARylation inhibition when MDA-MB-231 cells were treated with 500 nM of doxorubicin (Fig. S5A). However, we noticed that MDA-MB-231 cells were more prone to necrosis when PJ-34 and doxorubicin were combined than when the cells were treated with only one of these two drugs (Figs. S5B and S5C). Thus, even though the PJ-34 effects are moderate in MDA-MB-231 cells, PARP-1 inhibitors could be efficiently combined with other anti-cancer drugs to enhance the efficacy of the treatment.

### Accumulation of Ets-1 mediated by PARylation inhibition increases DNA damage

**Figure 6:**
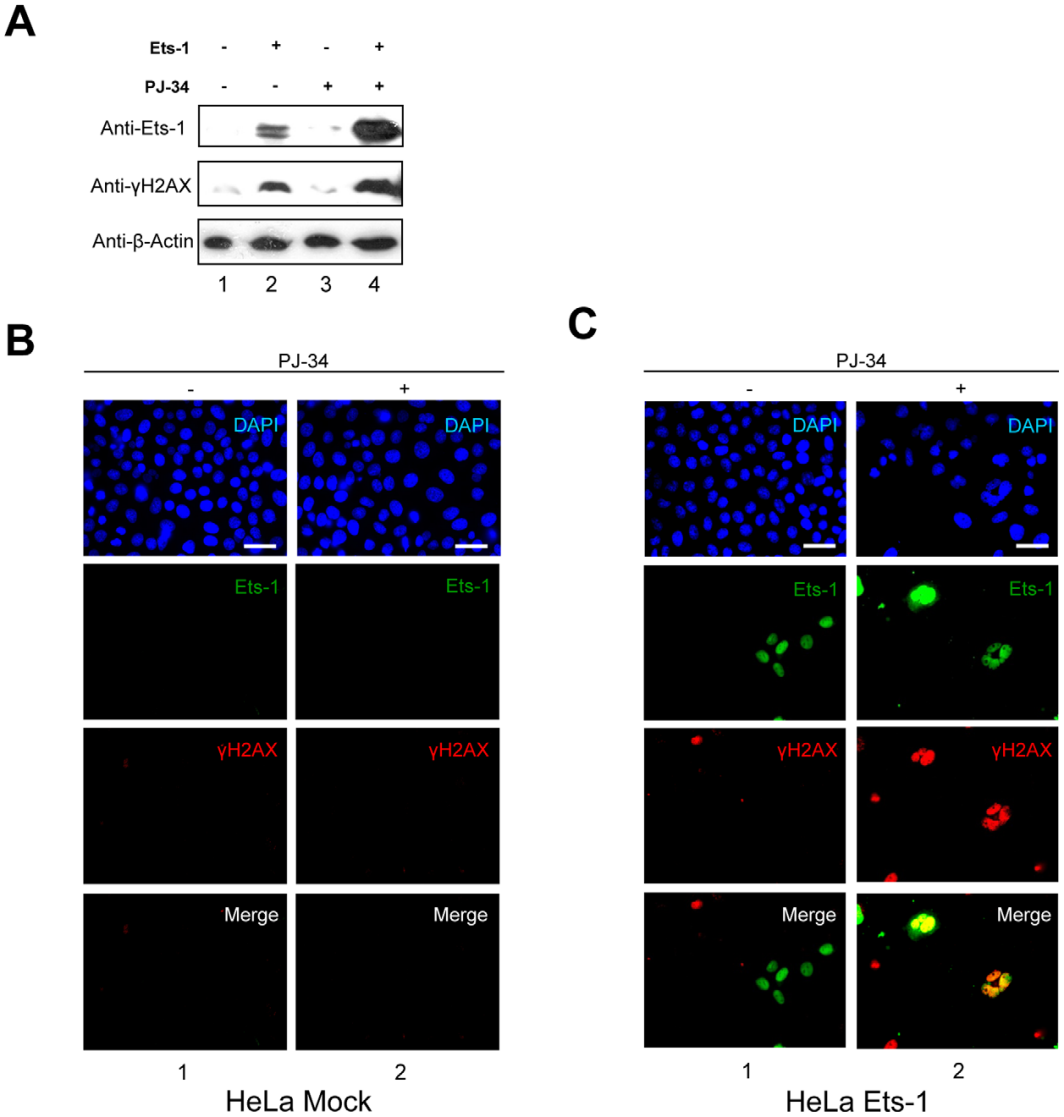
Accumulation of Ets-1 mediated by PARP-1 catalytic inhibition increases DNA damage. (**A**) Western blot analysis of phosphorylated H2AX (γH2AX) in transfected HeLa cells treated with PJ-34. pcDNA3 expression vectors without insert (lanes 1 and 3) and encoding Ets-1 (lanes 2 and 4) were transfected in HeLa cells at 50–80% confluence. At 24 h after transfection, cells were treated for 20 h with PJ-34 (10 µM; lanes 3 and 4). Cell lysates (30 µg total proteins) were analysed by Western blot using the C-20 anti-Ets-1 antibody and the anti-γH2AX antibody. (**B**) Immunofluorescence of γH2AX in HeLa cells treated with PJ-34. HeLa cells were treated with PJ-34 (10 µM) for 20 h. Ets-1 is visualised in green (Alexa Fluor® 488) and γH2AX in red (Alexa Fluor® 594). (**C**) Immunofluorescence of γH2AX and Ets-1 in transfected HeLa cells treated with PJ-34. HeLa cells were transfected with peGFP-C1-Ets-1 expression vector (1 µg) and treated with PJ-34 for 20 h. γH2AX is visualised in red (Alexa Fluor® 594) and Ets-1 in green (eGFP). In (B) and (C), nuclei were visualised with DAPI staining. Cells were examined by fluorescence microscopy at ×40 magnification. Scale bar = 20 µm.

**Figure 7:**
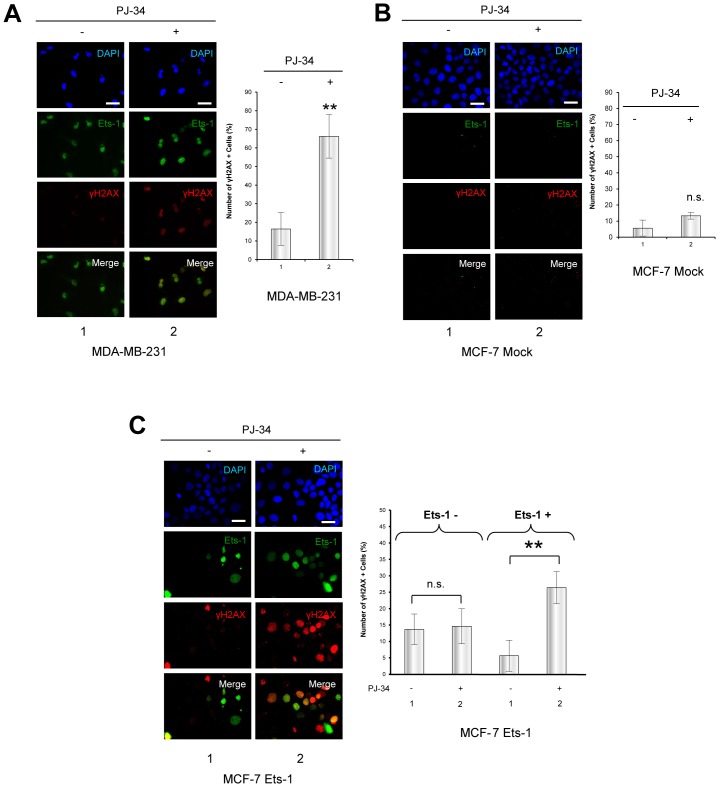
Ets-1 expression causes DNA damage mediated by PARP-1 catalytic inhibition in breast cancer cells. (**A**) Immunofluorescence of γH2AX in MDA-MB-231 cells after treatment with PJ-34. MDA-MB-231 cells were treated with PJ-34 (10 µM) for 20 h. Ets-1 is visualised in green (Alexa Fluor® 488) and γH2AX in red (Alexa Fluor® 594). Nuclei were visualised with DAPI staining. Cells were examined by fluorescence microscopy at ×40 magnification. Scale bar = 20 µm. Statistical analysis was performed by counting and examining after immunofluorescence more than 100 cells from three different experiments. γH2AX + indicates cells with more than 10 γH2AX foci in their nucleus (Fig. S6). All MDA-MB-231 cells are positive for Ets-1 expression (Ets-1 +). In the graph, the percentage of γH2AX + cells is given. ** p<0.01. (**B**) Immunofluorescence of γH2AX in MCF-7 cells after treatment with PJ-34. MCF-7 cells were treated with PJ-34 (10 µM) for 20 h. Ets-1 is visualised in green (Alexa Fluor® 488) and γH2AX in red (Alexa Fluor® 594). Statistical analysis was performed by counting and examining after immunofluorescence more than 100 cells from three different experiments. All MCF-7 cells are negative for Ets-1 expression (Ets-1 -). In the graph, the percentage of γH2AX + cells is shown. (**C**) Immunofluorescence of γH2AX and Ets-1 in Ets-1-transfected MCF-7 cells after treatment by PJ-34. MCF-7 cells were transfected with peGFP-C1-Ets-1 expression vector (1 µg) and treated with PJ-34 for 20 h. γH2AX is visualised in red (Alexa Fluor® 594) and Ets-1 in green (eGFP). Statistical analysis was performed as described in (A). In the graph, the percentage of γH2AX + cells is shown for the Ets-1 – and Ets-1 + cell populations. ** p<0.01. In (A) and (B), nuclei were visualised with DAPI staining. Cells were examined by fluorescence microscopy at ×40 magnification. Scale bar = 20 µm.

We then investigated the possibility that cell necrosis mediated by the inhibition of PARP-1 concomitant with Ets-1 accumulation was due to an increase in DNA damage as observed with Ets fusion proteins in prostate cancer and Ewing's sarcoma [26], [30]. To do so, we assessed the level of a histone mark of DNA double-strand breaks, phosphorylated H2AX (γH2AX) foci. Addition of PJ-34 to HeLa cells transfected with an empty vector did not have any effect on γH2AX in Western blot analysis (Fig. 6A, lane 3). Ets-1 expression was sufficient to observe an increase in the γH2AX signal (Fig. 6A, lane 2), but interestingly, the addition of PJ-34 led to a greater γH2AX signal along with Ets-1 accumulation (Fig. 6A, lane 4). To demonstrate that an increase in DNA damage was found only in Ets-1-expressing cells, we carried out immunofluorescence experiments using an anti-γH2AX antibody with HeLa cells transfected with or without eGFP-Ets-1. Results showed that non-transfected HeLa cells did not accumulate γH2AX foci after a 20 h treatment with PJ-34 (Fig. 6B). In GFP-Ets-1-transfected HeLa cells, γH2AX-positive cells did not particularly co-localise with eGFP-Ets-1 expression without PARylation inhibition (Fig. 6C, lane 1). However, addition of PJ-34 increased the number of γH2AX foci, but also made them much more prominent in eGFP-Ets-1-expressing HeLa cells (Fig. 6C, lane 2). We then examined the sensitivity of human breast cancer cells to DNA damage under PARylation inhibition with or without endogenous expression of Ets-1. Using immunofluorescence, we compared the accumulation of γH2AX foci after PJ-34 treatment in MDA-MB-231 cells (Fig. 7A), which endogenously express Ets-1, and in another breast cancer cell line, MCF-7 cells (Fig. 7B and 7C), which do not. Results showed that the addition of PJ-34 greatly increased the number of γH2AX foci in MDA-MB-231 cells (Fig. 7A, left panel, lane 2). Statistical analysis on these cells revealed a significant 4-fold increase in γH2AX-positive cells (>10 γH2AX foci per cell (Fig. S6)) in the presence of PJ-34 (Fig. 7A, graph, ** p<0.01), whereas it was not significant in MCF-7 cells (Fig. 7B, left panel and graph). This increase in γH2AX levels along with Ets-1 accumulation was also demonstrated by Western Blot in MDA-MB-231 cells treated with PJ-34, 5-AIQ and ABT-888 (Fig. S3). Then, we performed immunofluorescence with MCF-7 cells transfected with a vector expressing eGFP-Ets-1 (Fig. 7C, left panel). PJ-34 dramatically increased the number of γH2AX foci in MCF-7 cells transfected with eGFP-Ets-1 (Fig. 7C, left panel, lane 2). Statistical analysis of these experiments indicates that the addition of PJ-34 did not significantly increase the number of γH2AX-positive MCF-7 cells without Ets-1 expression (Fig. 7C, graph, Ets-1 -). In contrast, the number of γH2AX-positive cells was significant in Ets-1-positive MCF-7 cells with a 4.7-fold increase (Fig. 7C, graph, Ets-1 +, ** p<0.01). Interestingly, this is comparable to the 4-fold of increase observed in MDA-MB-231 cells. Thus, Ets-1 expression sensitises cancer cells to DNA damage when PARylation is inhibited.

## Discussion

Over the past few years, PARP-1 has received a great amount of attention, due to the emergence of PARP-1 inhibitors as new therapeutics in cancer. PARP-1 inhibitors have been shown to be effective in selectively inducing synthetic lethality in cancer cells, particularly in BRCA1- or BRCA2-deficient tumours [38]. Furthermore, PARP-1 inhibitors are considered as an effective clinical complement to chemotherapy, notably in triple-negative breast cancer [25]. Nevertheless, recent studies have questioned the use of certain PARP-1 inhibitors and underlined the disparity of effectiveness among inhibitors [39], [40]. Moreover, there is a lack of specificity with respect to targeting cancer cells versus normal cells, unless the tumours have already been sensitised to PARP-1 inhibition due to a deficient DNA repair system or specific genetic background [41]. For this reason, the greatest remaining challenge for the use of PARP-1 inhibitors is to find biomarkers that would indicate whether cancer cells would be sensitive to this type of treatment. Recent studies suggest that ETS-fusion proteins are good biomarkers of sensitivity to PARP-1 inhibitors [26], [30]. For example, EWS-Fli1 expression has been linked to sensitivity to PARP-1 inhibitors in a systematic identification of genomic markers of drug sensitivity in cancer cells [31].

**Figure 8:**
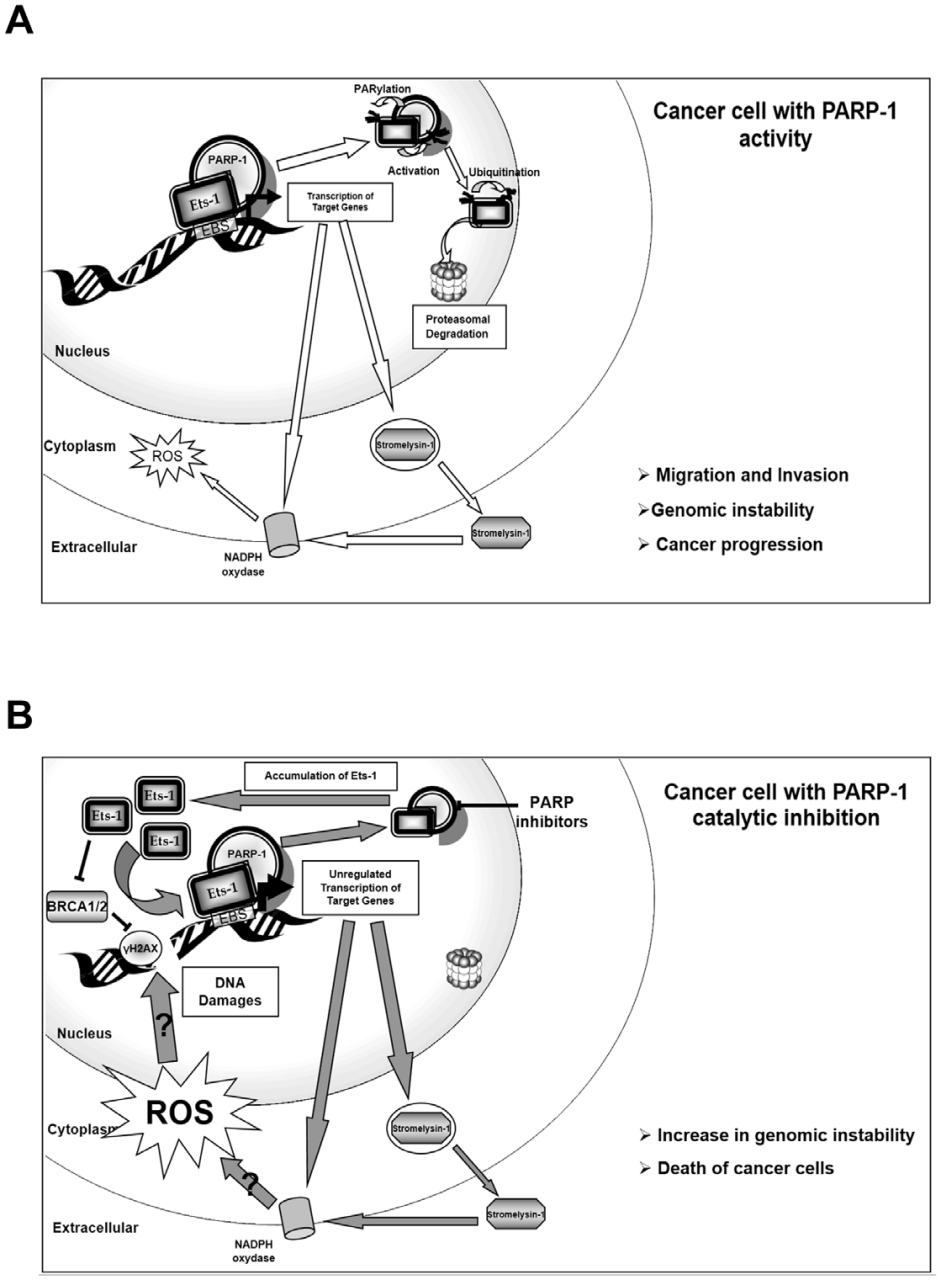
Proposed model for PARP-1 regulation of Ets-1 protein levels. (**A**) Regulation of Ets-1 protein levels by PARP-1 in cancer cells with PARylation activity. First, PARP-1 interacts with Ets-1 and allows Ets-1 to have full transcriptional activity. Second, Ets-1 activates PARP-1 catalytic activity and is then PARylated. PARylation of Ets-1 promotes its degradation by the proteasome, thereby allowing the cancer cells to modulate Ets-1 protein levels. The stimulation of the NADPH oxidase complex by Ets-1 transcriptional activity, both directly and indirectly by the matrix metalloprotease stromelysin-1, is also represented. This complex will generate reactive oxygen species (ROS). These ROS promote genomic instability, cancer progression and processes such as cancer cell migration and invasion. (**B**) Deregulation of Ets-1 protein levels in cancer cells under PARylation inhibition. Ets-1 is still able to interact with PARP-1 and has full transcriptional activity. However, Ets-1 is no longer PARylated and is therefore not targeted for proteasomal degradation. Thus, Ets-1 will accumulate in cancer cells and an increase in Ets-1 results in higher Ets-1 transcriptional activity, promoting the production of more ROS. High levels of ROS will generate a substantial increase in DNA damage (indicated by γH2AX) that cannot be repaired by PARP-1 that is inhibited, nor by BRCA1/2 because Ets-1 represses their expression. DNA damage accumulation, eventually, lead to the death of cancer cells.

In this study, we demonstrated a functional interaction between PARP-1 and Ets-1. We revealed an uncommon process whereby Ets-1 activates PARP-1 by direct interaction and is then PARylated in a DNA-independent manner. This suggests that Ets-1 PARylation does not depend on nicked DNA: the Ets-1/PARP-1 complex is probably not involved in DNA repair, but more likely in transcription mechanisms. In effect, we showed that PARP-1 is a dual regulator of Ets-1 transcriptional activity on the stromelysin-1 promoter. On the one hand, a PARP-1 knock-out drastically decreased the transactivation ability of Ets-1. On the other hand, inhibition of PARylation strongly increased Ets-1 activity on the same promoter. This seemingly paradoxical interaction has already been described for other factors such as AP-2, or TLE1 [24], [42]. In fact, PARP-1 is essential for recruiting the transcription machinery, such as the mediator complex or co-regulators (e.g. p300), although its catalytic activity is not required in these processes [22], [43]. Since p300 is also necessary for Ets-1-mediated transcription [44], we assume that Ets-1 fails to recruit certain positive co-regulators in the absence of PARP-1 and thus, that PARP-1 may be essential for Ets-1 on its target promoters (Fig. 8), as shown for Erg transcriptional activity [30]. In contrast, PARylation is known to negatively regulate certain proteins by modulating their affinity for DNA, by dissociating protein complexes or by promoting their ubiquitination and their proteasomal degradation [24], [42], [45]. E3 ligases, such as RNF146, can interact with PARylated proteins that activate its ubiquitin ligase activity to target them for proteasomal degradation [46]. Here, we demonstrated that PARylation inhibition causes a strong accumulation of Ets-1 in cancer cells with the same kinetics as those of Ets-1 accumulation under proteasome inhibition. Furthermore, we showed that the short isoform, Ets-1 p27, which is not sensitive to proteasomal degradation, does not accumulate in the presence of PARylation inhibitors. Thus, PARylation of Ets-1, which is due to its interaction with PARP-1, negatively regulates Ets-1, probably by promoting its ubiquitination and then its proteasomal degradation (Fig. 8A). Finally, we demonstrated that Ets-1 accumulation subsequent to PARylation inhibition sensitises cancer cells and leads to their death. The combination of Ets-1 accumulation and PARP-1 inhibition caused a strong increase in DNA damage only in Ets-1-expressing cells. ETS proteins are known to indirectly induce DNA double-strand breaks, although the precise mechanism is still unresolved [47]. Furthermore, recent studies have shown that TMPRSS2:Erg and EWS-Fli1 fusion proteins also sensitise cancer cells to PARP-1 inhibitors by inducing DNA damage, although these studies did not investigate the accumulation of these proteins [30], [31]. A very recent study showed that Ets-1 is a negative regulator of *BRCA1* and *BRCA2* gene expression. Knock-down of Ets-1 leads to increased BRCA1/2 expression, limiting the sensitivity to PARP-1 inhibitors [48]. If Ets-1 down-regulates BRCA1/2, this could prevent the homologous recombination DNA-repair pathway thus leaving only PARP-1 to repair DNA damage during DNA replication [49]. Moreover, it is known that Ets-1 is involved in reactive oxygen species (ROS) production, particularly through the transactivation of the *p47phox* gene, which is part of the NADPH oxidase complex, one of the major sources of ROS [50]. Furthermore, stromelysin-1 activates another member of the NADPH oxidase complex, the guanosine triphosphatase Rac1 [51]. In cancer, ROS production promotes migration and invasiveness as well as genomic instability (Fig. 8A) [51]. Under PARP-1 catalytic inhibition, Ets-1 is no longer PARylated and, therefore, is not directed to proteasomal degradation. As a result, Ets-1 accumulates in cancer cells and the increase in Ets-1 results in higher Ets-1 transcriptional activity, which may then stimulate ROS production by promoting NADPH oxidase complex activity, both directly and indirectly, particularly by increasing the expression of stromelysin-1 (Fig. 8B). Excessive oncogenic-induced ROS production by the NADPH complex is known to generate DNA damage with deleterious consequences for the cellular outcome [52]. Therefore, accumulated Ets-1 may induce numerous DNA lesions possibly through ROS production (Fig. 8B). Furthermore, without PARylation activity, PARP-1 cannot regulate Ets-1 protein levels, nor counteract the genotoxicity of ROS production and prevent cancer cell death [53].

In conclusion, we provide evidence that Ets-1 may be a potential new candidate as a biomarker of cancer cell sensitivity to PARP-1 inhibition. Beyond prostate cancer and Ewing sarcoma, PARP-1 inhibitors could be used to selectively kill Ets-1-expressing tumours in numerous cancers such as breast, lung, colorectal or ovarian carcinomas. Furthermore, our results give new insight into Ets-1 regulation in cancer cells and its link to DNA-repair proteins.

## Materials and Methods

### Plasmids

The construction of pcDNA3-Ets-1, pcDNA3-Ets-1p27, peGFP-C1-Ets-1p27 and human *stromelysin-1* promoter pGL3 reporter vectors have been previously described [33]. To produce the peGFP-C1-Ets-1 vector, human *ets-1* cDNA, obtained by PCR amplification (using the following primers: 5′-ATTTAAAGATCTAAGGCGGCCGTCGATCTC-3′ and 5′-TATGCGGCCGCTCACTCGTCGGCATCTGG CTTGAC-3′), was cloned in the *Bgl*II site of the peGFP-C1 expression vector (Clontech, Mountain View, CA, USA).

### Expression and purification of biotinylated recombinant Ets-1 protein

Biotinylated Ets-1 protein was produced and purified as described previously [32], using the principle of the T7-Impact™ System (New England Biolabs, Evry, France) adapted to induce biotinylation. To remove all trace of DNA, DNase I treatment was performed before purification [32].

### Cell culture

Human MDA-MB-231, MG63, MCF-7 and HeLa cells (ATCC, Manassas, VA, USA) as well as mouse 3T3 PARP-1 WT and PARP-1 KO cells [54] were cultured in Dulbecco's modified Eagle's medium (Invitrogen, Life Technologies, Saint Aubin, France) supplemented with 10% foetal bovine serum and 50 µg/ml gentamycin.

### Chemicals

We used three different PARP-1 inhibitors, PJ-34 (N-(6-oxo-5,6-dihydrophenanthridin-2-yl)-(N,N-dimethylamino) acetamide hydrochloride) (Enzo Life Sciences, Villeurbanne, France), 5-AIQ (5-aminoisoquinolinone hydrochloride) (Sigma–Aldrich, Lyon, France) and ABT-888 also known as veliparib ((R)-2-(2-methylpyrrolidin-2-yl)-1H-benzo[d]imidazole-4-carboxamide) (Selleckchem, Houston, TX, USA). MG-132 was purchased from Calbiochem (Merck-Millipore, Lyon, France) and doxorubicin from Sigma-Aldrich.

### Nuclear extract preparation

Cells were harvested and pellets were washed with cold PBS solution and then suspended in buffer-A (10 mM Hepes pH 7.9; 1.5 mM MgCl_2_; 10 mM KCl; 0.5 mM dithiothreitol (DTT); protease inhibitor cocktail (Roche Diagnostics GmbH)). The cell suspension was kept on ice for 10 min and vortexed to obtain cell nuclei. After centrifugation, the nuclear pellets were resuspended in buffer-B (20 mM Hepes pH 7.9; 1.5 mM MgCl_2_; 25% glycerol; 0.2 mM EDTA; 420 mM NaCl; 0.5 mM DTT; protease inhibitor cocktail) for 20 min at 4°C. The homogenates were centrifuged at 20,000 g for 5 min and supernatants were stored at −80°C. For DNase I treatment, MDA-MB-231 nuclear extracts were adjusted to 150 mM NaCl in buffer-B without NaCl or EDTA and supplemented with 7 mM MgCl_2_. DNase I was added to a final concentration of 50 µg/ml and samples were incubated at 4°C for 40 min.

### Streptavidin pull-down

Streptavidin sepharose™ beads (Amersham Biosciences, GE Healthcare, Velizy-Villacoublay, France) (30 µl) were washed with PBS and blocked by incubating for 1 h at 4°C in PBS with 1 mg/ml bovine serum albumin (Sigma–Aldrich). Biotinylated Ets-1 proteins (50 µg) were incubated with the prepared streptavidin sepharose™ beads for 30 min at 4°C under constant rotation. Beads were then washed with buffer containing 20 mM Hepes pH 7.9; 200 mM NaCl; 20% glycerol; 0.25 mM EDTA; 0.2% Igepal and 0.2 mM PMSF. The Ets-1 loaded beads were then incubated for 1 h at 4°C with 1.5 mg of pre-cleared nuclear extracts from MDA-MB-231, treated or not with DNase I, adjusting to 150 mM NaCl in buffer-B without NaCl. After extensive washing, bound proteins were eluted by boiling for 5 min in Laemmli buffer (50 mM Tris pH 6.8; 2% SDS; 5% β-mercaptoethanol; 10% glycerol; 0.1% bromophenol blue) and resolved on 10% SDS–PAGE followed by colloidal blue staining (Invitrogen).

### Mass spectrometry analysis

Protein bands of interest were excised from the gel and subjected to trypsin digestion as described previously [55]. The trypsin digests were then analysed by matrix-assisted laser desorption/ionisation-time of flight (MALDI-TOF) mass spectrometry on a Voyager-DE STR (Applied Biosystems) as described in reference [55]. The data were analysed by the ProFound search programme.

### Western blot analysis and antibodies

Western blot analyses were performed according to Baillat *et al.* [34]. Primary antibodies used were rabbit C-20 and mouse C-4 anti-Ets-1, rabbit H-250 anti-PARP-1, mouse F-2 anti-PARP-1, H-79 anti-c-Jun, DO-1 anti-p53 and C-14 anti-Erk-2 from Santa Cruz Biotechnology (Heidelberg, Germany), 10HA anti-PAR from Trevigen (Gaithersburg, MD, USA), (Ser139) 20E3 anti-phospho-H2AX from Cell Signaling (Danvers, MA, USA) and ab8227 anti-β-Actin from Abcam (Paris, France).

### Co-immunoprecipitation assay

Nuclear extracts from MDA-MB-231 or MG-63 cells (1 mg of proteins), treated with DNase I and diluted to a final concentration of 150 mM NaCl in buffer-B without NaCl, were pre-cleared with 4 µg of normal rabbit IgG-agarose conjugate (Santa Cruz) for 1 h at 4°C with rotation. The supernatant was then incubated with 10 µg of C-20 anti-Ets-1 or 10 µg of H-250 anti-PARP-1-agarose conjugate (Santa Cruz) overnight at 4°C with rotation. After centrifugation, agarose beads were washed four times with 150 mM NaCl buffer-B, resuspended in 20 µl of Laemmli buffer and heated at 95°C for 5 min. The supernatant was analysed by Western blot using the various antibodies.

### *In vitro* interaction assay

Streptavidin sepharose™ beads (Amersham Biosciences) (30 µl) were prepared as for the streptavidin pull-down assay. Biotinylated full-length Ets-1 protein (5 µg) or biotinylated Ets-1 p27 protein (2.5 µg) were incubated with the prepared streptavidin sepharose™ beads for 30 min at 4°C under constant rotation. The Ets-1 loaded beads were then incubated for 1 hour at 4°C with 500 ng of recombinant PARP-1 protein (Trevigen). After extensive washing, bound proteins were eluted by boiling for 5 min in Laemmli buffer and analysed by Western blot.

### *In vitro* PARylation assay

PARylation of Ets-1 proteins *in vitro* by PARP-1 was assayed using recombinant human PARP-1 enzyme, high specific activity grade (Trevigen). Briefly, 1 µg of Ets-1 or 500 ng of Ets-1 p27 was incubated with 500 ng of PARP-1 for 20 min at 37°C in the presence or absence of sonicated linear double-stranded DNA (salmon sperm) in reaction buffer (20 mM Hepes pH 7.9; 1.5 µM ZnCl_2_; 100 mM NaCl; 10 mM MgCl_2_; 10% Glycerol; 1 mM DTT; 0.5 mM β-NAD^+^ (Sigma-Aldrich)). In absence of linear double-stranded DNA, samples were treated with 1 U of Benzonase® to prevent presence of DNA or RNA (Merck-Millipore, Lyon, France). Reactions were stopped by boiling in Laemmli buffer. PARylation was monitored by Western blot using anti-PAR antibody (Trevigen).

### Retroviral infection and PARylation in cells

Retrovirus production, infection using empty pLPCX-SBP-6His or pLPCX-SBP-6His-Ets-1 and cell selection were performed as previously described [33]. To investigate PARylation of Ets-1 in cells, HeLa cell nuclear extracts were prepared and SBP-6His-Ets-1 was purified on streptavidin beads. After extensive washing, bound Ets-1 proteins were eluted by boiling for 5 min in Laemmli buffer and analysed by Western blot for PARylation.

### Transfection and reporter gene assay

Transfection and reporter gene assays were performed as described previously [18]. Transfections were performed with the human *stromelysin-1* promoter pGL3 reporter vector and increasing amounts of PJ-34 were added in the presence or absence of the pcDNA3-Ets-1 expression vector. pcDNA3-PARP-1 vector was used for the rescue experiment in PARP-1 KO cells.

### Immunofluorescence

Immunofluorescence experiments were performed as described previously [33], using rabbit anti-Ets-1 antibody (C-20) or mouse anti-Ets-1 antibody (C-4) or/and mouse anti-PARP-1 antibody (F-2) or mouse anti-phospho-H2AX (20E3) as primary antibodies and goat anti-rabbit Alexa Fluor® 594 or goat anti-mouse Alexa Fluor® 488 antibodies (Invitrogen) as secondary antibodies. Nuclei were counterstained with 2 µg mL^−1^ 4,6-diamino-2-phenylindole (DAPI) fluorescent dye. Fluorescent signals of Alexa Fluor® 594, 488 and eGFP were examined using an Eclipse 80i microscope (Nikon Instruments, Kingston, United Kingdom).

### Time-lapse imaging experiments

For eGFP-Ets-1 accumulation, time-lapse imaging experiments were carried out with a Leica AF600 LX microscope. HeLa cells were grown on 35 mm Petri dish containing a 14 mm glass microwell (MatTek Corporation, Ashland, MA, USA) and were then transiently transfected with vectors. After addition of PJ-34 (10 µM) or MG-132 (5 µM) (Calbiochem, Merck-Millipore, Lyon, France), cells were observed for 12 h in a temperature-controlled chamber (37°C) with a CO_2_ supply. Analysis was performed with LAS AF (Leica Microsystèmes, Nanterre, France) imaging software. For the survival assay, time-lapse imaging experiments were carried out with a BioStation IM-Q (Nikon Instruments). HeLa or MDA-MB-231 cells were seeded in a four-well chambered Hi-Q4 dish (Nikon Instruments). HeLa cells were then transiently transfected with empty pcDNA3 or pcDNA3-Ets-1 vectors. After addition of Hoechst 33242 (0.5 µg/mL, Molecular Probes, Life Technologies, Saint Aubin, France) and propidium iodide (PI) (1 µg/mL, Sigma-Aldrich), cells were monitored for 20 h in a temperature-controlled chamber (37°C) with a CO_2_ supply. The proliferation/necrosis rate was determined using ImageJ software (Rasband, W.S., ImageJ, U. S. National Institutes of Health, Bethesda, Maryland, USA, http://imagej.nih.gov/ij/, 1997–2012.). Briefly, segmentation was performed on both Hoechst (total number of cells) and PI (necrotic cells) channels. An automated particle analysis algorithm was then used to calculate the total number of necrotic and non-necrotic cells. Data were then normalised on the initial number of cells and used to calculate a mean proportion of necrosis. Means and standard deviations were calculated from at least 6 fields per acquisition condition.

### Flow cytometry analysis

HeLa cells were grown until they reached 70% confluence in 6-well plates before transfection with empty pcDNA3 or pcDNA3-Ets-1 vectors. After incubation with PARP-1 inhibitors for 20 h, dead floating cells as well as trypsinised adherent cells were harvested, centrifuged and resuspended in PBS buffer to a final concentration of 5×10^5^ cells/mL. Cells were then incubated with PI (final concentration of 5 µg/mL in PBS) for 15 min in the dark and analysed by flow cytometry (CYAN, Beckman Coulter, Villepinte, France) using Summit analysis software.

## Supporting Information

Figure S1 **MALDI-TOF spectrum of PARP-1 purified by streptavidin pull-down.**
(TIF)

Figure S2 **Sub-localisation of Ets-1 and PARP-1 in MDA-MB-231 cells by immunofluorescence.** Ets-1 is visualised in red (Alexa Fluor® 594), and PARP-1 in green (Alexa Fluor® 388). The insets are close-ups of the boxed cells. Nuclei were visualised using DAPI stain. Cells were examined under a fluorescence microscope at ×40 magnification. Scale bar = 20 µm.
(TIF)

Figure S3 **Effect of PARP-1 catalytic inhibition on the level of Ets-1 and γH2AX.** MDA-MB-231 cells were treated with PJ-34 (1 µM), 5-AIQ (1 µM) or ABT-888 (1 µM) for 20 h. Cell lysates (30 µg total proteins) were analysed by Western blot using different antibodies (see Materials and Methods) against Ets-1, γH2AX, p53 and β-Actin.
(TIF)

Figure S4 **PARP-1 catalytic inhibition using ABT-888 leads to cancer cell death by necrosis.** (**A**) Time-lapse imaging experiments. HeLa cells were grown in Hi-Q4 dishes until 70% confluence and transfected with empty pcDNA3 (250 µg; left panel) or pcDNA3-Ets1 (250 µg; right panel) vectors 24 h before being treated with ABT-888 (1 µM) or left untreated. Cells were stained with Hoechst 33242 (blue) and PI (red) for live-cell imaging and monitored for 20 h. Scale bar = 20 µM. (**B**) Graphical representation of the proportion of necrotic HeLa cells (%) at three time points (see Materials and Methods). (**C**) Flow cytometry cell-death detection: HeLa cells were grown in 6-well plates until 70% confluence and transfected with pcDNA3 (1 µg; left panel) or pcDNA3-Ets1 (1 µg; right panel) vectors for 24 h and left untreated (dashed lines) or treated with ABT-888 (solid lines) for an additional 20 h incubation. Necrotic cell death was then determined by flow cytometry after PI staining. Numbers under the horizontal bar represent the percentages of specific ABT-888-induced necrotic cell death in each condition. Flow cytometry profiles shown are representative of three replicate experiments.
(TIF)

Figure S5 **Effect of PJ-34 and Doxorubicin on the MDA-MB-231 cells survival.** (**A**) MDA-MB-231 cells were treated with PJ-34 (10 µM) and/or doxorubicin (500 nM) for 20 h. Cell lysates (30 µg total proteins) were analysed by Western blot using an anti-Ets-1 antibody (C-20).(**B**) Time-lapse imaging experiments of MDA-MB-231 cells treated with PJ-34 and doxorubicin. MDA-MB-231 cells were grown in Hi-Q4 dishes until 80% confluence, treated with doxorubicin (500 nM) and treated with PJ-34 (10 µM) or left untreated. Cells were stained with Hoechst 33242 (blue) and PI (red) for live-cell imaging and monitored for 20 h. Scale bar = 20 µM. (**C**) Graphical representation of the proportion of necrotic MDA-MB-231 cells (%) at three time points to summarise results from Fig. 5D and from (B).
(TIF)

Figure S6 **Determination of γH2AX-positive cells for statistical analyses.** γH2AX-positive cells were determined by counting γH2AX foci, visualised here in red (Alexa Fluor® 594), in the cell nucleus from immunofluorescence experiments. Cells with no or less than 10 γH2AX foci were considered to be negative (γH2AX −; 1 and 2); while cells with more than 10 γH2AX foci were considered to be positive (γH2AX +; 3 and 4).
(TIF)
