# Supplementary figures and images for: The Level of Ets-1 Protein Is Regulated by Poly(ADP-Ribose) Polymerase-1 (PARP-1) in Cancer Cells to Prevent DNA Damage
Source: PLoS One. 2013 Feb 6;8(2):e55883. doi: 10.1371/journal.pone.0055883 (PMC3566071; doi:10.1371/journal.pone.0055883)

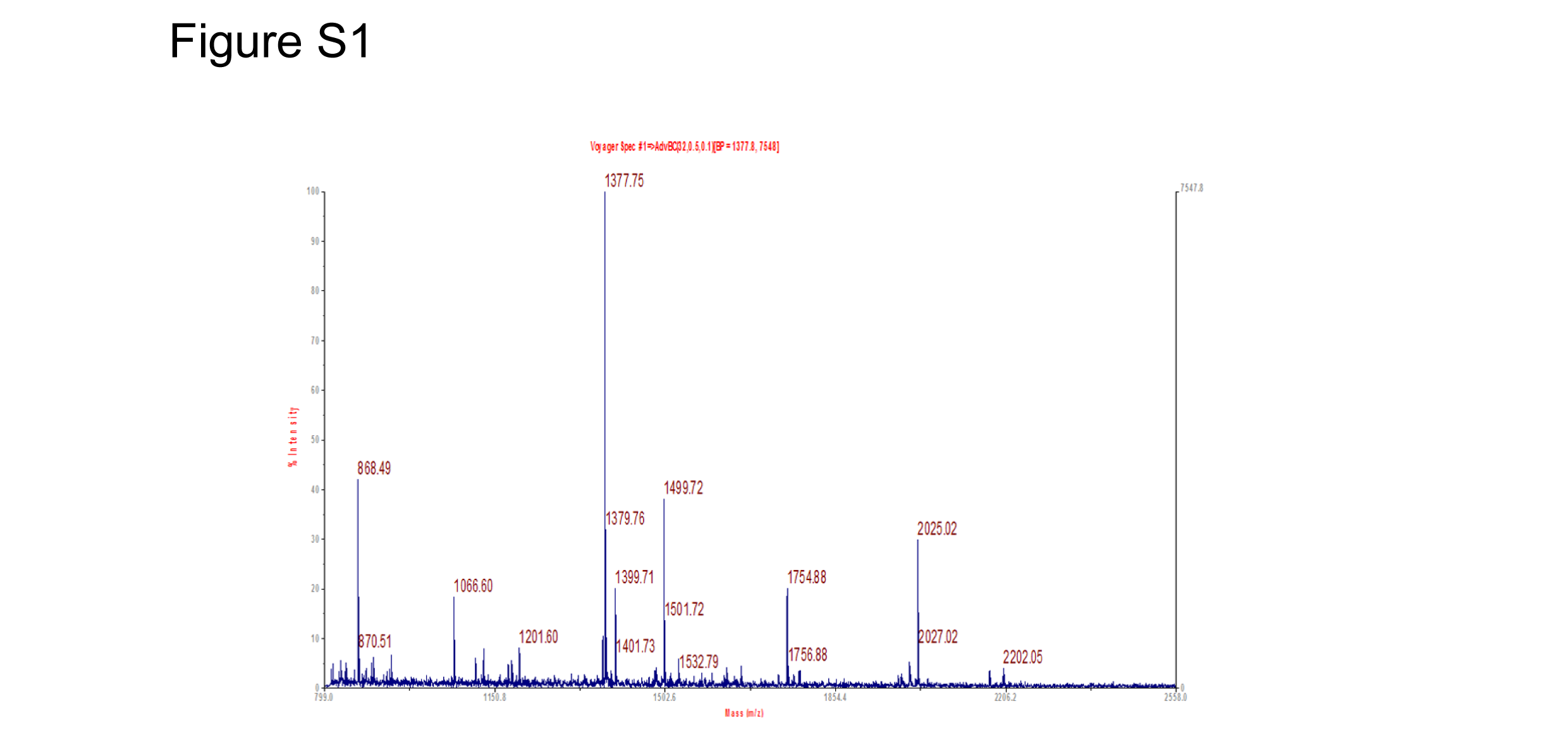

Supplement: Figure S1 — MALDI-TOF spectrum of PARP-1 purified by streptavidin pull-down. (TIF) [file pone.0055883.s001.tif]

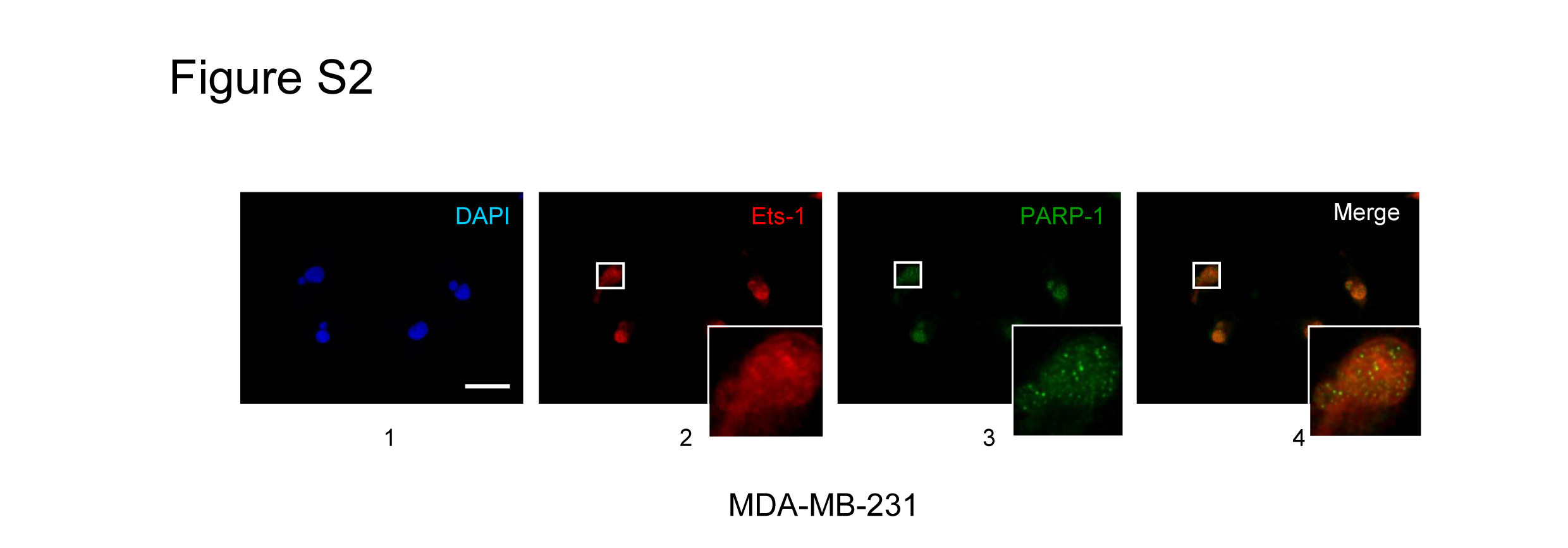

Supplement: Figure S2 — Sub-localisation of Ets-1 and PARP-1 in MDA-MB-231 cells by immunofluorescence. Ets-1 is visualised in red (Alexa Fluor® 594), and PARP-1 in green (Alexa Fluor® 388). The insets are close-ups of the boxed cells. Nuclei were visualised using DAPI stain. Cells were examined under a fluorescence microscope at ×40 magnification. Scale bar = 20 µm. (TIF) [file pone.0055883.s002.tif]

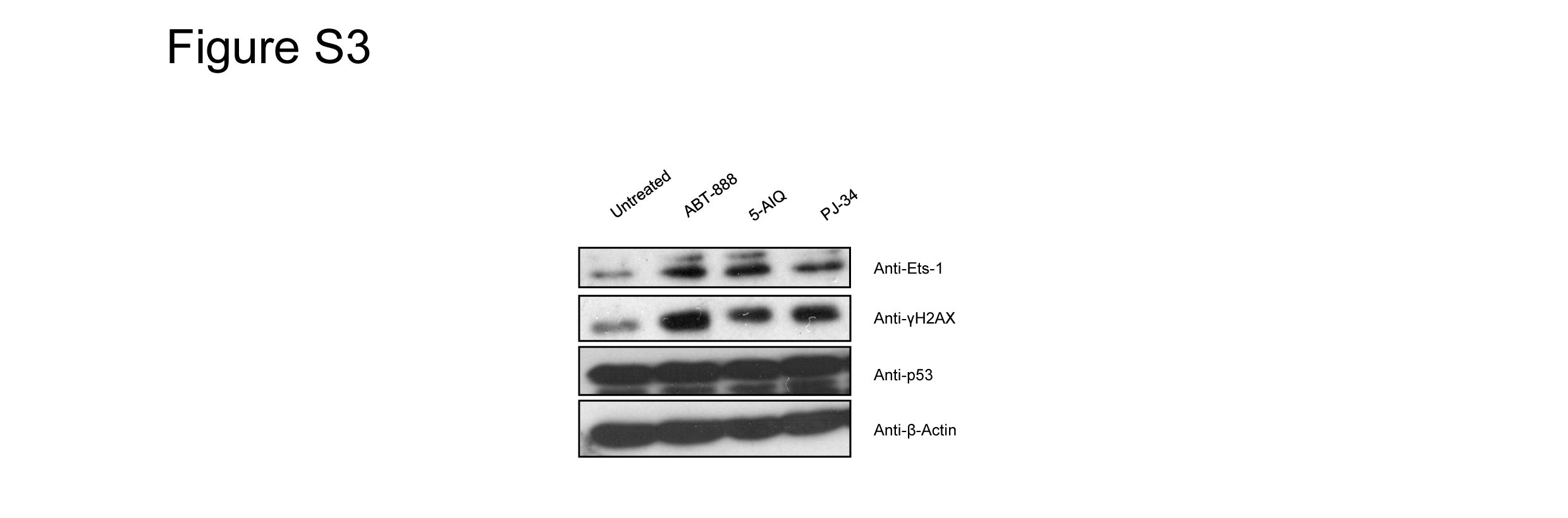

Supplement: Figure S3 — Effect of PARP-1 catalytic inhibition on the level of Ets-1 and γH2AX. MDA-MB-231 cells were treated with PJ-34 (1 µM), 5-AIQ (1 µM) or ABT-888 (1 µM) for 20 h. Cell lysates (30 µg total proteins) were analysed by Western blot using different antibodies (see Materials and Methods) against Ets-1, γH2AX, p53 and β-Actin. (TIF) [file pone.0055883.s003.tif]

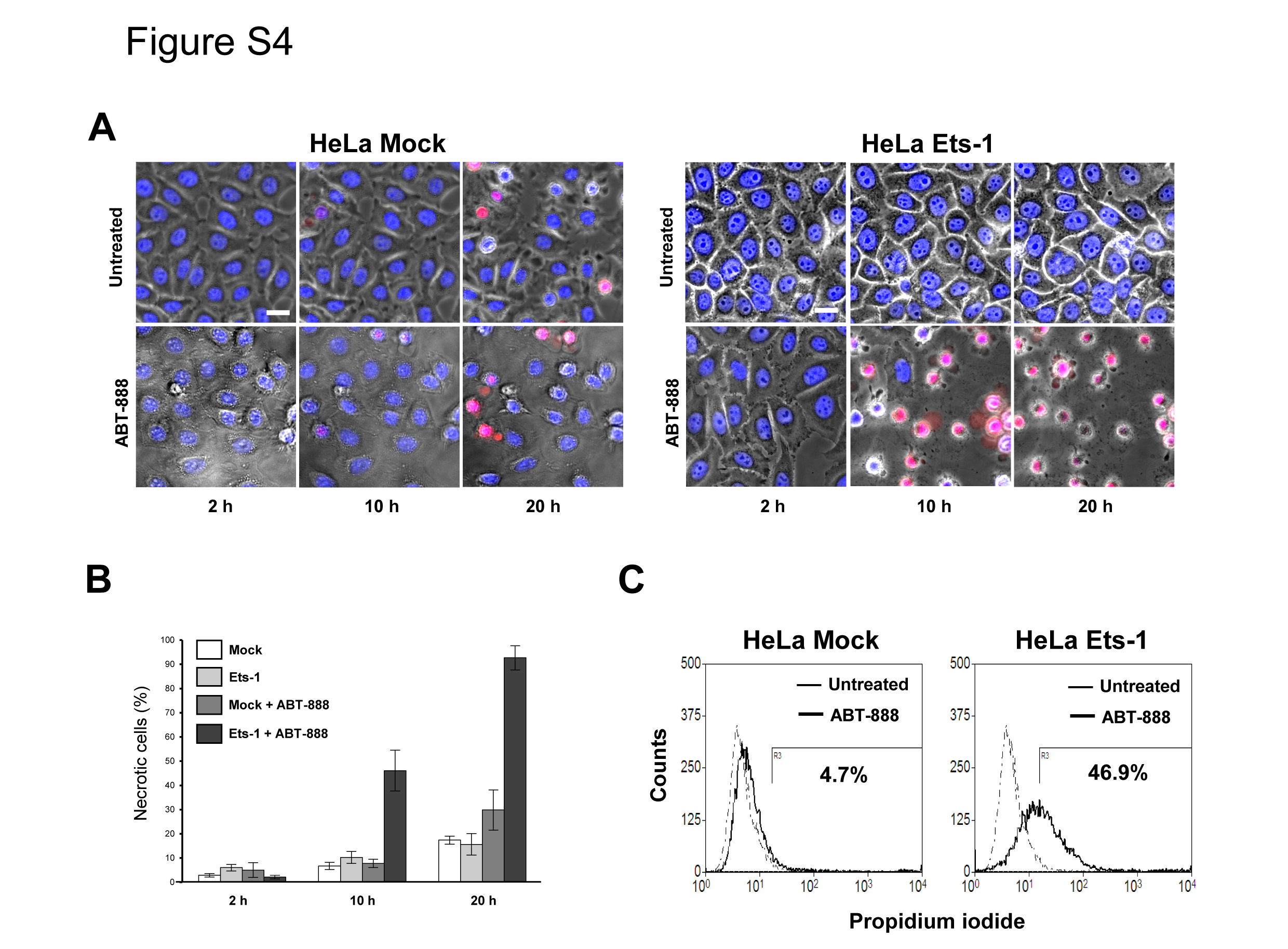

Supplement: Figure S4 — PARP-1 catalytic inhibition using ABT-888 leads to cancer cell death by necrosis. (A) Time-lapse imaging experiments. HeLa cells were grown in Hi-Q4 dishes until 70% confluence and transfected with empty pcDNA3 (250 µg; left panel) or pcDNA3-Ets1 (250 µg; right panel) vectors 24 h before being treated with ABT-888 (1 µM) or left untreated. Cells were stained with Hoechst 33242 (blue) and PI (red) for live-cell imaging and monitored for 20 h. Scale bar = 20 µM. (B) Graphical representation of the proportion of necrotic HeLa cells (%) at three time points (see Materials and Methods). (C) Flow cytometry cell-death detection: HeLa cells were grown in 6-well plates until 70% confluence and transfected with pcDNA3 (1 µg; left panel) or pcDNA3-Ets1 (1 µg; right panel) vectors for 24 h and left untreated (dashed lines) or treated with ABT-888 (solid lines) for an additional 20 h incubation. Necrotic cell death was then determined by flow cytometry after PI staining. Numbers under the horizontal bar represent the percentages of specific ABT-888-induced necrotic cell death in each condition. Flow cytometry profiles shown are representative of three replicate experiments. (TIF) [file pone.0055883.s004.tif]

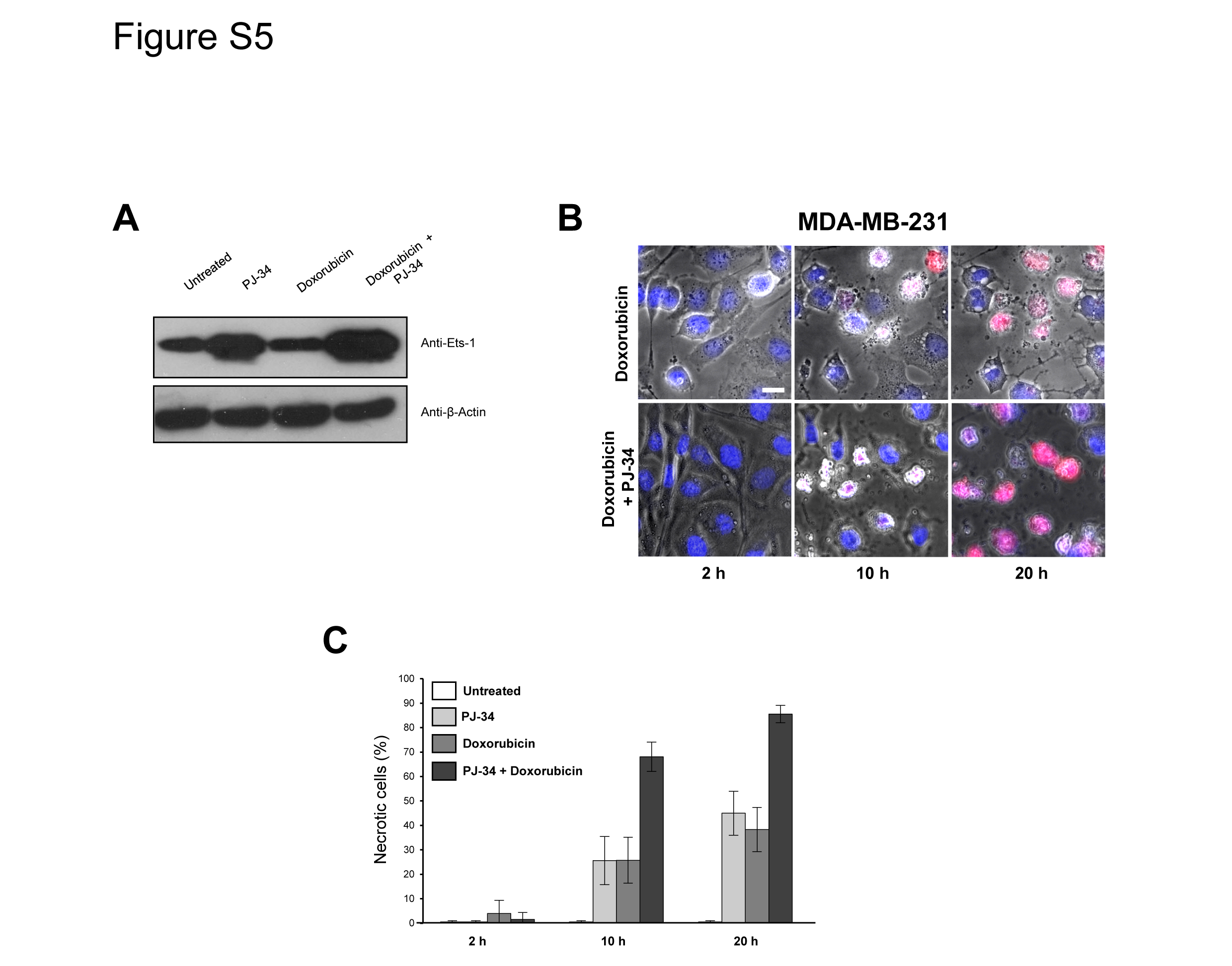

Supplement: Figure S5 — Effect of PJ-34 and Doxorubicin on the MDA-MB-231 cells survival. (A) MDA-MB-231 cells were treated with PJ-34 (10 µM) and/or doxorubicin (500 nM) for 20 h. Cell lysates (30 µg total proteins) were analysed by Western blot using an anti-Ets-1 antibody (C-20).(B) Time-lapse imaging experiments of MDA-MB-231 cells treated with PJ-34 and doxorubicin. MDA-MB-231 cells were grown in Hi-Q4 dishes until 80% confluence, treated with doxorubicin (500 nM) and treated with PJ-34 (10 µM) or left untreated. Cells were stained with Hoechst 33242 (blue) and PI (red) for live-cell imaging and monitored for 20 h. Scale bar = 20 µM. (C) Graphical representation of the proportion of necrotic MDA-MB-231 cells (%) at three time points to summarise results from Fig. 5D and from (B). (TIF) [file pone.0055883.s005.tif]

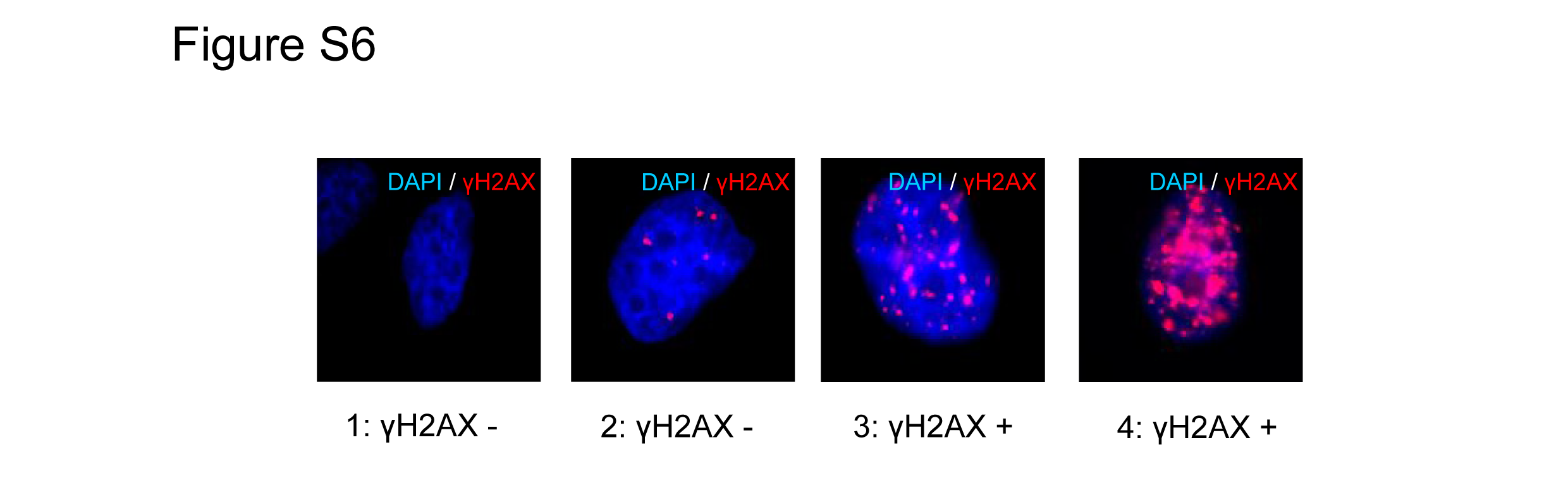

Supplement: Figure S6 — Determination of γH2AX-positive cells for statistical analyses. γH2AX-positive cells were determined by counting γH2AX foci, visualised here in red (Alexa Fluor® 594), in the cell nucleus from immunofluorescence experiments. Cells with no or less than 10 γH2AX foci were considered to be negative (γH2AX −; 1 and 2); while cells with more than 10 γH2AX foci were considered to be positive (γH2AX +; 3 and 4). (TIF) [file pone.0055883.s006.tif]
